# Analgesic and Anticancer Activity of Benzoxazole Clubbed 2-Pyrrolidinones as Novel Inhibitors of Monoacylglycerol Lipase

**DOI:** 10.3390/molecules26082389

**Published:** 2021-04-20

**Authors:** Obaid Afzal, Abdulmalik Saleh Alfawaz Altamimi, Mir Mohammad Shahroz, Hemant Kumar Sharma, Yassine Riadi, Md Quamrul Hassan

**Affiliations:** 1 Department of Pharmaceutical Chemistry, College of Pharmacy, Prince Sattam Bin Abdulaziz University, Al Kharj 11942, Saudi Arabia; y.riadi@psau.edu.sa; 2 Department of Pharmaceutical Chemistry, College of Pharmacy, Sri Satya Sai University of Technology and Medical Sciences, Sehore 466001, Madhya Pradesh, India; mirshahroz@gmail.com (M.M.S.); hkspharma@rediffmail.com (H.K.S.); 3 Department of Pharmacology, School of Pharmaceutical Education and Research, Jamia Hamdard, New Delhi 110062, India; quamrulhassan309@gmail.com

**Keywords:** analgesic, anticancer, pyrrolidin-2-one, benzoxazole, MAGL inhibitors, molecular docking

## Abstract

Ten benzoxazole clubbed 2-pyrrolidinones (**11**–**20**) as human monoacylglycerol lipase inhibitors were designed on the criteria fulfilling the structural requirements and on the basis of previously reported inhibitors. The designed, synthesized, and characterized compounds (**11**–**20**) were screened against monoacylglycerol lipase (MAGL) in order to find potential inhibitors. Compounds **19** (4-NO_2_ derivative) and **20** (4-SO_2_NH_2_ derivative), with an IC_50_ value of 8.4 and 7.6 nM, were found most active, respectively. Both of them showed micromolar potency (IC_50_ value above 50 µM) against a close analogue, fatty acid amide hydrolase (FAAH), therefore considered as selective inhibitors of MAGL. Molecular docking studies of compounds **19** and **20** revealed that carbonyl of 2-pyrrolidinone moiety sited at the oxyanion hole of catalytic site of the enzyme stabilized with three hydrogen bonds (~2 Å) with Ala51, Met123, and Ser122, the amino acid residues responsible for the catalytic function of the enzyme. Remarkably, the physiochemical and pharmacokinetic properties of compounds **19** and **20**, computed by QikProp, were found to be in the qualifying range as per the proposed guideline for good orally bioactive CNS drugs. In formalin-induced nociception test, compound **20** reduced the pain response in acute and late stages in a dose-dependent manner. They significantly demonstrated the reduction in pain response, having better potency than the positive control gabapentin (GBP), at 30 mg/kg dose. Compounds **19** and **20** were submitted to NCI, USA, for anticancer activity screening. Compounds **19** (NSC: 778839) and **20** (NSC: 778842) were found to have good anticancer activity on SNB-75 cell line of CNS cancer, exhibiting 35.49 and 31.88% growth inhibition (% GI), respectively.

## 1. Introduction

**Figure 1:**
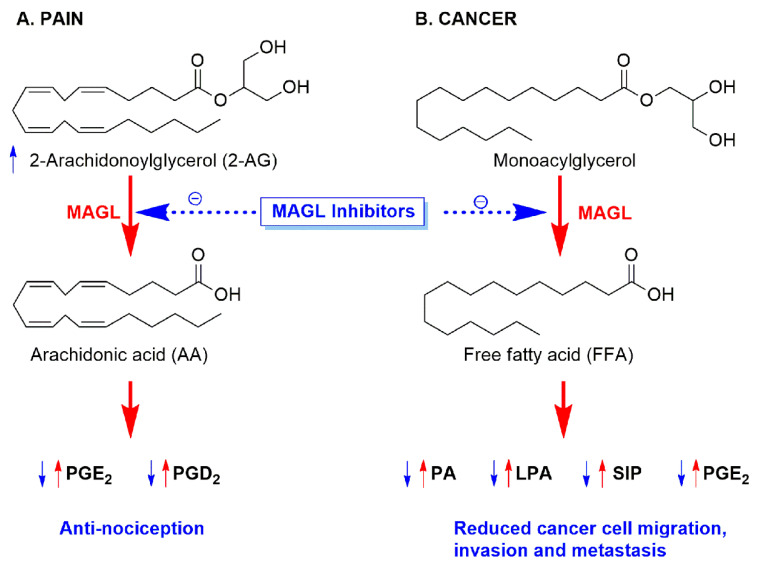
Role of MAGL inhibitors in the alleviation of pain and cancer. PG: prostaglandins; PA: phosphatidic acid; LPA: lysophosphatidic acid; and S1P: sphingosine-1-phosphate.

Endocannabinoids (endogenous ligands), cannabinoid (CB) receptors, and proteins for their biological synthesis and degradation constitute the endocannabinoid system (ECS) [1]. Endocannabinoids are biosynthesized from the membrane phospholipids [2]. Endocannabinoid, *N*-arachidonoyl ethanolamine (AEA, Anandamide) functions as partial agonist on CB_1_ and CB_2_ receptors. It has low affinity for CB_2_ and moderate affinity for CB_1_. Endocannabinoids, 2-arachidonoylglycerol (2-AG) function as full agonist and have moderate affinity for both the receptors. Interestingly, 2-AG is the major endocannabinoid and is found to be approximately 170-fold higher in concentration than AEA, in the brain [3]. AEA and 2-AG hydrolysis and degradation are facilitated by fatty acid amide hydrolase (FAAH) and monoacylglycerol lipase (MAGL) enzymes, correspondingly [1]. MAGL (an α/β-hydrolase) hydrolyzes 2-AG into glycerol and free fatty acid (FFA), by the action of the catalytic triad organized with Ser122, Asp239 and His269 amino acids in the active site of the enzyme [4]. MAGL hydrolyzes approximately 85% of total 2-AG in the CNS [5] and generates arachidonic acid (AA), giving rise to neuro-inflammatory PGE_2_ and PGD_2_ prostaglandins [6]. MAGL inhibitors ameliorates neuropathic pain by increasing 2-AG and decreasing neuro-inflammatory prostaglandins in the CNS (Figure 1) [7]. In addition, 2-AG demonstrated analgesic activity by acting on CB_1_ receptors in the CNS and periphery [8,9,10,11]. It is reported that JJKK-048 (MAGL, IC_50_ 363 pM) exhibited analgesic activity in tail immersion and writhing test [12]. MAGL inhibition has shown significant neuroprotective and anti-inflammatory potential in Parkinson’s and Alzheimer’s disease [13,14]. PF-06795071 (IC_50_ 3 nM), a MAGL inhibitor, is reported to have considerable anti-neuroinflammatory potential [15]. Another MAGL inhibitor, ABX-1431 (IC_50_ 14 nM) is under clinical trials for broad range of CNS disorders like Tourette syndrome [16]. Many more research findings encouraged that the inhibitors of MAGL has therapeutic potentials in pain and CNS disorders [17,18,19,20].

The governing importance of MAGL in abnormal lipolysis in cancer has been demonstrated [21]. MAGL was originally known for its lipolytic action on monoacylglycerols from stored triacylglycerols into glycerol and free fatty acids (FFA) [22]. Cancer cells utilizes this lipolytic pathways for their hastened proliferation [23]. The cancer-supporting action of MAGL is due to elevated FFA levels. This MAGL-FFA pathway promotes in vivo tumor growth by increasing FFA-derived oncogenic signaling lipids (PA, LPA, S1P, and PGE_2_) [18]. These protumorigenic lipid mediators encourage tumor growth, angiogenesis, and metastasis in cancer (Figure 1) [24]. MAGL is reported to expressed vastly in aggressive type of cancer cells and is associated with pathogenesis, proliferation, and in vivo tumor growth. MAGL inhibition disrupts cancer cell proliferation, growth and metastasis [25,26,27]. The anticancer effect of MAGL inhibition in prostate cancer was totally abolished by cotreatment with SR141716 (rimonabant; CB_1_ receptor antagonist) and fatty acids, signifying that amplified endocannabinoid action and reduced stock of FFA from MAGL inhibition is the reason behind antitumor effect [26].

MAGL inhibitors identified till date includes URB602 (IC_50_ 28 µM) [28], CAY10499 (IC_50_ 144 nM) [29], SPB01403 (IC_50_ 31 µM) [30], JZL184 (IC_50_ 8 nM) [31], SAR629 (IC_50_ 1.1 nM) [32], KML29 (IC_50_ 3.6 nM) [33], ML30 (IC_50_ 0.54 nM) [34], JJKK-048 (IC_50_ 363 pM) [35], ABX1431 (IC_50_ 14 nM) [16], R(3t) (IC_50_ 3.6 nM) [36], PF-06795071 (IC_50_ 3 nM) [15], a benzoylpiperidine derivative (IC_50_ 80 nM) [37], and a benzisothiazolinone derivative (IC_50_ 34.1 nM) [38]. JJKK-048 and KML29 both were reported to be highly selective MAGL inhibitors, and their selectivity is more than 10,000-fold over FAAH. [33,35]. In addition, 3D crystal structures of human MAGL enzyme were elucidated by X-ray crystallography, and are available on protein data bank [32,36,39,40]. A comprehensive description of MAGL crystal structures and inhibitors were reviewed [41]. Our group has also identified nanomolar MAGL inhibitors, viz. ZINC24092691 (IC_50_ 10 nM), ZINC12863377 (IC_50_ 39 nM), a ZINC24092691 analogue (IC_50_ 6.5 nM), a thiazole-5-carboxylate derivative (IC_50_ 37 nM), and a pyrrolidin-2-one linked benzimidazole derivative (compound **25**; IC_50_ 9.4 nM) [42,43,44,45]. In continuation of our work on MAGL inhibitors, we have designed novel 2-pyrrolidinone linked benzoxazole derivatives and screened them for analgesic and anticancer effects.

## 2. Results

**Figure 2:**
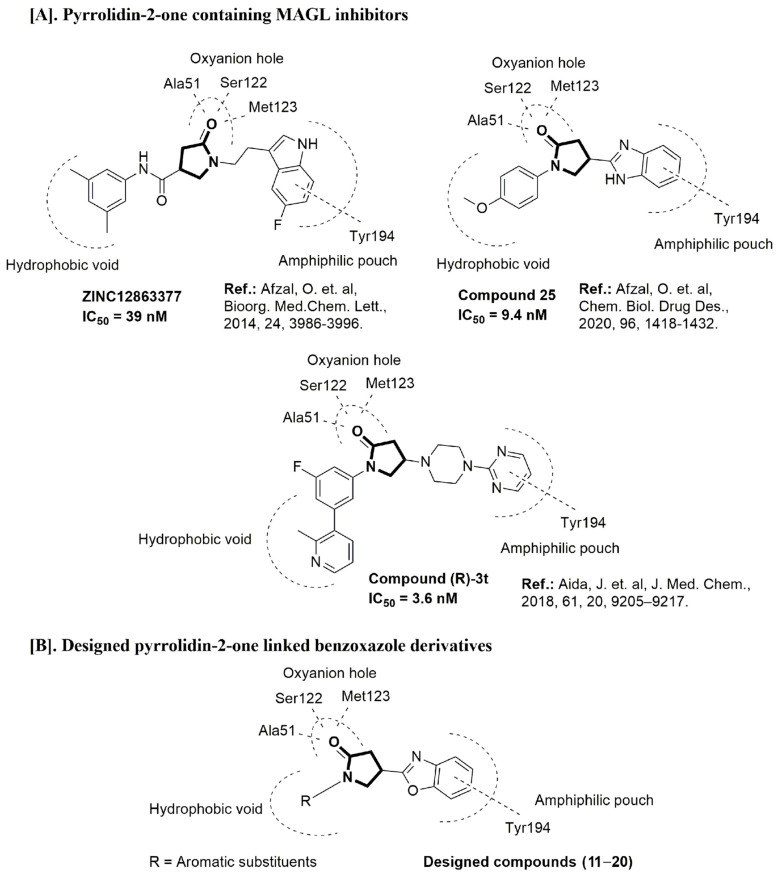
Design of novel pyrrolidin-2-one linked benzoxazole MAGL inhibitors. (**A**) Binding pattern of reported pyrrolidin-2-one MAGL inhibitors; ZINC12863377 [42], compound 25 [45] and (R)-3t [36]. (**B**) Designed compounds (**11**–**20**).

**Figure 3:**
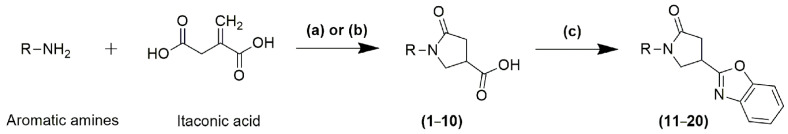
Scheme for synthesis of the intermediates (**1**–**10**) and target compounds (**11**–**20**). (**a**) Reflux in water (Method 1); (**b**) Fusion at 130–140 °C (Method 2); (**c**) 2-aminophenol, polyphosphoric acid, 150–160 °C, sodium carbonate.

The binding pattern of pyrrolidin-2-one derivatives (ZINC12863377, compound **25** and compound R-3t), and the basic structural requirement for MAGL inhibitors was kept in mind to design novel pyrrolidin-2-one linked benzoxazole derivatives (Figure 2). The route of synthesis of compounds (**11**–**20**) is presented in Figure 3.

### 2.1. Chemistry 

Intermediate compound **1**, (*1-Benzyl-5-oxopyrrolidine-3-carboxylic acid*), was successfully prepared by fusion of benzylamine and methylidenesuccinic acid in water, while compounds **2–10** were synthesized as reported by us in our previous publication [45]. The fusion of synthesized 1-substituted-5-oxopyrrolidine-3-carboxylic acids (**1–10**) with 2-aminophenol was done by the procedure reported earlier with some minor modifications [46]. The fusion of acids (**1–10**) and 2-aminophenol was carried out by the use of polyphosphoric acid, giving better yields (57–70%) and purity of benzoxazole derivatives (**11**–**20**).

The prototype intermediate compound **1**, (*1-Benzyl-5-oxopyrrolidine-3-carboxylic acid*), revealed typical peaks at 1627 cm^−1^ (carbonyl of acid), 1734 cm^−1^ (carbonyl of pyrrolidinone), and 3241 cm^−1^ (O-H of acid) in IR spectrum. In ^1^H-NMR spectrum of compound **1**, two methyl protons were found resonating as singlet at δ 3.62. The multiplets resonating at δ 2.62–2.76 indicated two COCH_2_ protons, and at δ 4.29–4.44 they indicated two NCH_2_ and one CH proton of 2-pyrrolidinone ring. Five aromatic protons of the benzyl ring were found resonating at δ 7.20–7.36 as a multiplet. The typical singlet was assigned to COOH proton at δ 11.35 and was found to be D_2_O exchangeable. Final prototype compound **11** (4-(Benzo[d]oxazol-2-yl)-1-benzylpyrrolidin-2-one) exhibited specific IR bands at 1621 cm^−1^ (C=N of benzoxazole) and 1703 cm^−1^ (C=O of pyrrolidin-2-one). The two protons of CH_2_ of benzyl appeared at δ 3.95–4.01 as a multiplet. Two COCH_2_ protons of the 2-pyrrolidinone ring was assigned at δ 2.60–2.77 as a multiplet. The multiplets located at δ 3.13–3.18 and 3.81–3.86 were ascribed to one CH proton and two NCH_2_ protons of the 2-pyrrolidinone ring, correspondingly. Six aromatic protons were found resonating at δ 7.38–7.58 as a multiplet. The doublet resonating at δ 7.73–7.78 was assigned to the aromatic protons of benzoxazole. The other two protons of benzoxazole were found resonating at δ 7.94–7.96 as doublet. The M^+^ (molecular ion) peak of compound **11** was found at 292.14, validating its successful synthesis.

### 2.2. Human MAGL Assay

The assay was executed by Cayman’s assay kit by the reported procedure [29]. All the ten compounds (**11**–**20**) were screened for *h*MAGL inhibitory potential. The substituted phenyl derivatives (**13**–**20**) were established to reduce the MAGL activity at 100 μM concentration below 50%. Compound **19** (4-NO_2_ derivative) and compound **20** (4-SO_2_NH_2_ derivative) were the most potent, with an IC_50_ of 8.4 and 7.6 nM, correspondingly. The structure–function relationship of benzoxazole derivatives is displayed *h*MAGL inhibitory activity as follows: 4-SO_2_NH_2_ > 4-NO_2_ > 3-Cl,4-F > 4-OCH_3_ > 4-Cl > 4-OH > 4-CH_3_ > 2-CH_3_ > phenyl/benzyl. The IC_50_ of standard controls, selective MAGL inhibitors, CAY10499 (IC_50_ = 415 nM), and for JZL184 (IC_50_ = 10 nM) were comparable to the reported values [29]. The outcomes of the experiments are presented in Table 1.

### 2.3. Human FAAH Assay 

Derivatives having IC_50_ in nanomolar range (compound **15**, **16**, **18**, **19**, and **20**) were nominated for further screening against FAAH, an allied hydrolase of MAGL [47]. They displayed micromolar potency, with an IC_50_ value ranging from 25 to 68 µM against FAAH. The benzoxazole derivatives having 4-NO_2_ phenyl (**19**) and 4-SO_2_NH_2_ phenyl (**20**), with an FAAH IC_50_ value greater than 50 µM, were considered selective MAGL inhibitors. The IC_50_ of standard control, URB597 (selective FAAH inhibitor), was 5 nM, equivalent to the value reported (IC_50_ = 4.6 nM) [48]. The outcomes of the experiments are presented in Table 1.

### 2.4. Molecular Docking Study

**Figure 4:**
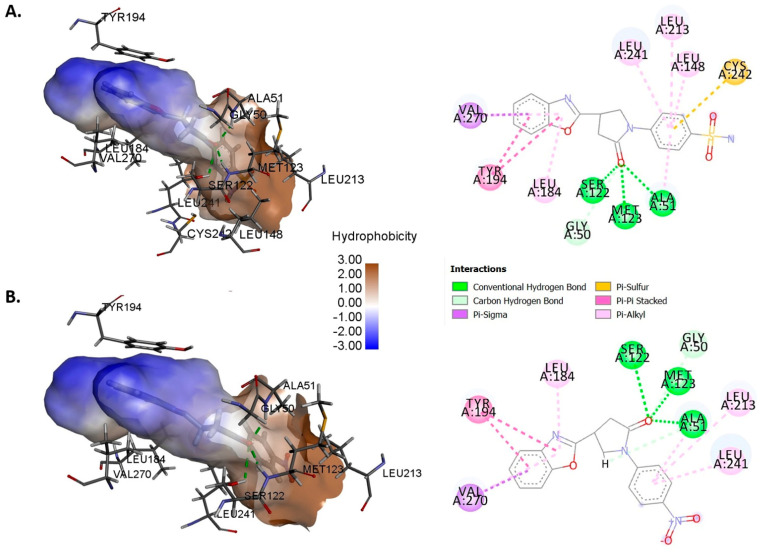
Glide XP docking; 3D and 2D representation of the binding pattern of compound **19** (**A**), and **20** (**B**) in the catalytic site of MAGL. The picture (2D and 3D) for the docked complexes were obtained from Discovery studio visualizer 2020. The hydrophobicity was calculated from the default option available.

Most active and selective compounds identified by MAGL and FAAH inhibition assay (**19** and **20**), were docked at the catalytic center of MAGL, in order to get an insight of their binding pattern, with the help of XP Glide docking using Maestro (Schrodinger). Compounds **19** and **20**, showed comparable docking scores of −9.87 and −9.83, respectively. The binding of compounds **19** and **20** in the active site of MAGL revealed that the carbonyl group of pyrrolidinone is located exactly in the oxyanion hole and stabilized by three hydrogen bonds (~2Å) with alanine 51, serine 122, and methionine 123. Serine 122 is one of the critical amino acid residues of the catalytic triad of MAGL. The benzoxazole moiety is found to be positioned in the amphiphilic pouch, having π-π stacking contact with the amino acid Tyr194. The 4-NO_2_ (**19**) and 4-SO_2_NH_2_ (**20**) phenyl ring of the ligands were involved in hydrophobic (van der Waals) attractions with the amino acids, leucine 148, 213, and 241. In addition, the 3D and 2D binding pattern of compounds **19** and **20** in the catalytic location of MAGL is depicted in Figure 4.

### 2.5. Pharmacokinetic and Physicochemical Characteristics

To investigate the potential of the identified derivatives (**19** and **20**) to cross the selectively permeable membranes of hematoencephalic barrier (BBB), to develop orally active CNS drugs, their pharmacokinetic and physicochemical features were computed by QikProp (ADMET predictor) of Schrodinger. Guidelines, concerning the validation and optimization of orally active CNS compounds, were developed by Ghose et. al., by analyzing 35 characteristic features of orally bioavailable 317 CNS and 626 non-CNS drugs [49]. This guideline states that in order to design high-quality CNS drugs, the molecule must qualify by the following parameters: TPSA less than 76 Å^2^ (ideally 25–60 Å^2^), number of N atoms between 1–2, comprising 1 aliphatic amine, 2–4 side chains on/outside rings, number of polar H atoms ˂ 3 (ideally 0–1), SASA 460–580 Å^2^, molecular volume 740–970 Å^3^, and must have +ve QikProp CNS property. Remarkably, most of the properties of compounds **19** and **20**, computed by QikProp, were found to be in the qualifying range as per the proposed guideline (Table 2). The properties of compound **19** were found to be within the qualifying range except dipole moment. For compound **20**, 5 out of 35 properties is just slightly above the upper qualifying limit. Most importantly, qualifying limits for CNS active drugs in terms of TPSA is from 3.8 to 109, and the calculated TPSA for compound **20** was found to be 119.08. Therefore, the designing of more potent MAGL inhibitors having physicochemical and pharmacokinetic properties within the preferred CNS limits is required. 

### 2.6. In Silico Absorption and Toxicity Profile

The selected compounds (**19** and **20**) were evaluated for their absorption and toxicity profile by a bioinformatics tool admetSAR [50]. The results suggested that both the compounds have high blood–brain barrier (BBB) penetration properties as well as high chance of human intestinal absorption. In AMES test, compound **19** was found to be mutagenic, while **20** was non-mutagenic. Carcinogenicity test revealed that both the compounds were non-carcinogens. The LD50 values in rat were also evaluated, a compound with high value is considered as less lethal. The LD50 for compounds **19** and **20** were found to be 2.30 and 2.21 mol/kg, respectively. Overall, compound **20** has better toxicity profile as compared to compound **19** (Table 3). 

### 2.7. Analgesic Activity

**Figure 5:**
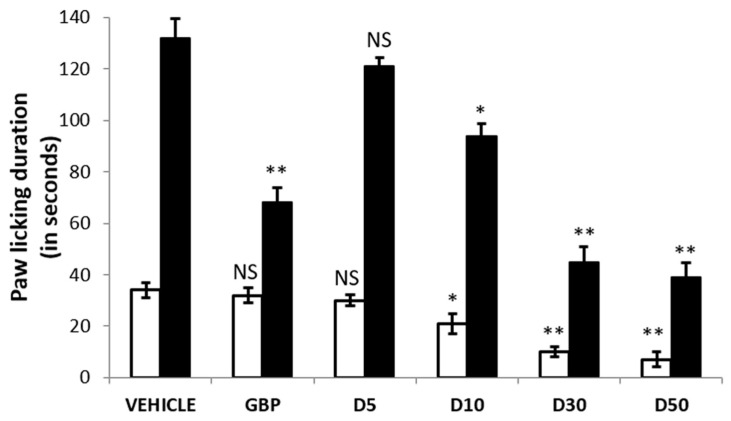
Formalin-induced analgesic test; a dose of the test compound **20** (5, 10, 30, and 50 mg/kg, p.o, suspended in 0.5% CMC) was administered 4 h before formalin injection (50 µL, 2.5%). Reference drug, gabapentin (100 mg/kg, i.p, dissolved in 0.9% normal saline) was injected 30 min before formalin injection. Total paw licking and biting duration, Stage I (white bar, 0–5 min) and stage II (black bar, 10–30 min) was recorded as a measure of pain behavior. Data is represented as mean ± SEM from a group of 10 animals. * *p* < 0.05, ** *p* < 0.01, *p* > 0.05 (NS, nonsignificant) vs. control (vehicle). GBP: Gabapentin; D5, D10, D30, andD50 are the dose concentrations of test compound **20**.

The formalin-induced analgesic test is an extensively acknowledged animal nociception model. In order to evaluate both central and peripheral effects of the compound (20), formalin-induced nociception model was selected for analgesic activity. The formalin induced behavioral response comprises two typical phases, stage I and II. Stage I persists up to five minutes after formalin injection and is characterized by acute pain with vigorous licking and biting of the injected site. Stage I consists the action formalin on afferent C-fiber nociceptors. While Stage II starts 10–30 min after formalin injection and persists till 60 min, characterized by periodic licking and biting of the injected site. Stage II imitates the action of central sensitization of the spinal dorsal horn neurons [51,52]. Compound **20** was selected for analgesic activity due to its higher potency (IC_50_ 7.6 nM). Compound **20** (suspensions prepared with 0.5% CMC) were administered per oral (p.o, in doses of 5, 10, 30, and 50 mg/kg body weight, 4 h prior to the formalin injection. Gabapentin (GBP), (dissolved in 0.9% normal saline), was chosen as positive control (reference drug) and administered intraperitoneal (i.p) in 100 mg/kg dose. GBP exhibited little analgesic effects in Stage I (acute nociception), in comparison to the control (0.5% CMC). Though, in Stage II, it displayed significant reduction of paw licking and biting, endorsing GBP central effects. However, compound **20**, reduced the pain response significantly both in acute (Stage I) and late (Stage II) phases, in a dose-dependent manner. They significantly demonstrated the reduction in pain response, having better potency than the positive control GBP at 30 mg/kg. The duration (in seconds) of paw licking and paw biting throughout Stage I and II is provided in Figure 5.

### 2.8. Anticancer Activity 

Compounds **19** and **20** were supplied to National Cancer Institute (USA), for sulforhodamine B (SRB) assay and anticancer screening [53,54]. Single-dose (10 µM) assay results for compounds **19** and **20** were provided as a mean of percent growth (% G) and growth inhibition (% GI) against 60 cell lines of nine types of cancers and are tabulated in Table 4. Derivatives **19** (NSC: 778839) and **20** (NSC: 778842) were found to have good anticancer activity towards SNB-75 cell line of CNS cancer, having % growth inhibition (% GI) of 35.49 and 31.88, respectively. Compound **20** showed 22.22 and 18.03% GI of HOP-92 and HOP-62 cell lines of non-small cell lung cancer, respectively. Both the compounds **19** and **20** were also found active on UO-31 renal cancer cell line with % GI of 21.18 and 29.95, respectively. Compound **19** showed % GI of 19.99 on T-47D, while compound **20** showed % GI of 19.89 on MDA-MB-231/ATCC cell lines of breast cancer.

## 3. Discussion

Ten benzoxazole clubbed 2-pyrrolidinone derivatives (**11**–**20**) as the inhibitors of monoacylglycerol lipase were designed on the criteria fulfilling the structural requirements and on the basis of previously reported inhibitors [36,42,43,44,45]. The designed, synthesized, and characterized compounds (**11**–**20**) were screened against monoacylglycerol lipase (MAGL) in order to find potential inhibitors. The substituted phenyl derivatives (**13**–**20**) were established to reduce the MAGL activity at 100 μM concentration below 50%. Compound **19** (4-NO_2_ derivative) and compound **20** (4-SO_2_NH_2_ derivative) were the most potent, with IC_50_ of 8.4 and 7.6 nM, correspondingly. The benzoxazole derivatives having 4-NO_2_ phenyl (**19**) and 4-SO_2_NH_2_ phenyl (**20**), with an FAAH IC_50_ value greater than 50 µM, were considered selective MAGL inhibitors. In molecular docking studies, compounds **19** and **20** showed comparable docking scores of −9.87 and −9.83, respectively. The binding of compounds **19** and **20** in the active site of MAGL revealed that the carbonyl group of pyrrolidinone is located exactly in the oxyanion hole and stabilized by three hydrogen bonds (~2 Å) with alanine 51, serine 122, and methionine 123. Serine 122 is one of the critical amino acid residues of the catalytic triad of MAGL. The benzoxazole moiety is found to be positioned in the amphiphilic pouch, having π-π stacking contacts with the amino acid tyrosine 194. The 4-NO_2_ phenyl (**19**) and 4-SO_2_NH_2_ phenyl (**20**) part of the ligand was engaged in hydrophobic (Van der Waals) attractions with the amino acids leucine 148, 213, and 241. The binding patterns of compounds **19** and **20** in the catalytic site of MAGL were found to be similar as those of the reported inhibitors bound crystal structures [32,36,39,41]. Remarkably, the physiochemical and pharmacokinetic properties of compounds **19** and **20** computed by QikProp were found to be in the qualifying range as per the proposed guideline for good orally bioactive CNS drugs. Moreover, compound **20** showed better toxicity profile than compound **19**, as predicted by admetSAR [50]. In formalin-induced analgesic test, compound **20** reduced the pain response significantly both in acute (stage I) and late (stage II) phases in a dose-dependent manner. It significantly demonstrated the reduction in pain response, having better potency than the positive control GBP, at the dose of 30 mg/kg. Moreover, in one dose (10 µM), anticancer screening by SRB assay, compounds **19** (NSC: 778839) and **20** (NSC: 778842) were found to have good anticancer activity towards SNB-75 cell line of CNS cancer, having % growth inhibition (% GI) of 35.49 and 31.88, respectively. Therefore, the present work concluded that compound **20** is the potential lead compounds that can be further manipulated at points 1 and 4 of the 2-pyrrolidinone moiety for the discovery and development of more selective and potent inhibitors of MAGL for neuropathic pain and CNS disorders including cancers.

## 4. Experimental

### 4.1. Chemistry

Reagents and solvents were procured from Merck Ltd. (New Delhi, India) and Sigma-Aldrich Ltd. (New Delhi, India). Progress and completion of the reactions was checked by thin-layer chromatography (TLC). Melting points of the derivatives were determined by open tube capillary method and uncorrected. Elemental analysis data were obtained from CHNOS elemental analyzer (Vario EL III, Elementar Analysensysteme GmbH, Langenselbold, Germany). Shimadzu FT-IR spectrometer (Shimadzu Analytical Pvt. Ltd., New Delhi, India) was used for recording IR spectrum (4000–400 cm^−1^), by preparing KBr pellets. ^1^H-NMR spectrum of the derivatives were obtained from Bruker 300 MHz NMR instrument (Bruker Avance AV-III type, Billerica, MA, USA) using CDCl_3_ or DMSO-*d*_6_ as solvent. ^1^H-NMR spectra of compounds **11**–**20** can be found in the Supplementary Material. Molecular mass (*m*/*z*) of the derivatives were obtained by UPLC-MS (Q-TOF-ESI) (Waters Corp., Milford, MA, USA). 

#### 4.1.1. Synthesis of 1-(Aryl Substituted)-5-Oxopyrrolidine-3-Carboxylic Acids (**1**–**10**)

##### Method-1 (for compound **1**)

Equimolar amount of benzylamine (50 mmol) and itaconic acid (50 mmol, 6.5 g) in 50 mL of tripled distilled water was refluxed for about 45–60 min. The contents were then chilled, filtered, and washed with cold water. The obtained solid was dissolved in minimum quantity of aq. NaOH (10%). After treatment with activated charcoal, the solution was filtered and acidified with dil. HCl in order to obtain the precipitate. The filtered solid was washed with cold water, dried, and purified by recrystallization from ethanol/water mixture. 

*1-Benzyl-5-Oxopyrrolidine-3-Carboxylic Acid* (**1**), white solid; yield: 75%; m.p. 142–145° C; IR: 1518 (C=C), 1627 (C=OOH), 1734 (C=O), 2947 (sp_3_ C-H), 3045 (Ar C-H), 3241 (COO-H); ^1^H-NMR (DMSO-*d*_6_) δ (ppm): 2.63–2.76 (m, 2H, COCH_2_), 3.62 (s, 2H, CH_2_), 4.29–4.44 (m, 3H, NCH_2_ and CH_pyrr_), 7.20–7.36 (m, 5H, Ar-H), 11.35 (s, 1H, COOH, D_2_O exchangeable); ESI-MS (*m*/*z*): 219.12 [M]^+^; Anal. calcd. For C_12_H_13_NO_3_: C, 65.74; H, 5.98; N, 6.39. Found: C, 65.80; H, 5.85; N, 6.50.

##### Method-2 (for compounds **2–10**)

Intermediate compounds (**2–10**) were synthesized as per the procedure reported in our previous publication [45].

#### 4.1.2. Synthesis of 4-(Benzoxazolyl)-1-(Aryl Substituted)Pyrrolidin-2-Ones (**11**–**20**) 

Appropriate 1-(aryl substituted)-5-oxopyrrolidine-3-carboxylic acids (20 mmol), 2-aminophenol (20 mmol, 2.18 g) and polyphosphoric acid (20 g) in an RBF were heated to 150–160° C and stirred for 2–3 h. The content of the RBF (round bottom flask) was cooled at RT; 5% NaCO_3_ (25 mL) was added and heated for 10 min. The content was chilled and transferred in a flask having 100 mL of water, and stirred at RT for 15 min. The filtered solid was washed three times with water (50 mL), dried, and purified by recrystallization with ethanol. The derivatives were then purified by chromatography using ethylacetate:hexane (1:4) as solvent.

*4-(Benzo[d]oxazol-2-yl)-1-benzylpyrrolidin-2-one* (**11**), pale-yellow solid; yield: 57%; m.p. 160–162 °C; IR: 1370 (C-N), 1555 (C=C), 1621 (C=N), 1703 (C=O), 3077 (Ar C-H); ^1^H-NMR (DMSO-*d*_6_) δ (ppm): 2.60–2.77 (m, 2H, COCH_2_), 3.13–3.18 (m, 1H, CH_pyrr_), 3.81–3.86 (m, 2H, NCH_2_), 3.95–4.01 (m, 2H, CH_2_), 7.38–7.58 (m, 6H, Ar-H), 7.73–7.78 (d, 1H, Ar-H, *J* = 15.6 Hz), 7.94–7.96 (d, 2H, Ar-H, *J* = 8.4 Hz); ESI-MS (*m*/*z*): 292.14 [M]^+^, 293.14 [M + H]^+^; Anal. calcd. for C_18_H_16_N_2_O_2_: C, 73.95; H, 5.52; N, 9.58. Found: C, 74.16; H, 5.70; N, 9.82.

*4-(Benzo[d]oxazol-2-yl)-1-phenylpyrrolidin-2-one* (**12**), pale-yellow solid; yield: 67%; m.p. 148–150 °C; IR: 1395 (C-N), 1552 (C=C), 1615 (C=N), 1698 (C=O), 3070 (Ar C-H); ^1^H-NMR (DMSO-*d*_6_) δ (ppm): 2.86–3.20 (m, 2H, COCH_2_), 4.16–4.40 (m, 3H, NCH_2_ and CH_pyrr_), 7.16–8.18 (m, 9H, Ar-H); ESI-MS (*m*/*z*): 278.13 [M]^+^, 279.13 [M + H]^+^; Anal. calcd. for C_17_H_14_N_2_O_2_: C, 73.37; H, 5.07; N, 10.07. Found: C, 73.60; H, 5.18; N, 10.33.

*4-(Benzo[d]oxazol-2-yl)-1-(o-tolyl)pyrrolidin-2-one* (**13**), pale-yellow solid; yield: 68%; m.p. 162–164 °C; IR: 1404 (C-N), 1568 (C=C), 1607 (C=N), 1691 (C=O), 3079 (Ar C-H); ^1^H-NMR (DMSO-*d*_6_) δ (ppm): 2.18 (s, 3H, CH_3_), 2.88–3.17 (m, 2H, COCH_2_), 4.12–4.18 (m, 1H, CH_pyrr_), 4.26–4.39 (m, 2H, NCH_2_), 7.18–7.21 (m, 2H, H-5_phenyl_ and H-6_phenyl_), 7.36–7.54 (m, 4H, H-5_benzoxazole_, H-6_benzoxazole_, H-3_phenyl_ and H-4_phenyl_), 7.98–8.01 (d, 1H, H-7_benzoxazole_, *J* = 8.1 Hz), 8.09–8.12 (d, 1H, H-4_benzoxazole_, *J* = 7.8 Hz); ESI-MS (*m*/*z*): 292.13 [M]^+^, 293.13 [M + H]^+^; Anal. calcd. for C_18_H_16_N_2_O_2_: C, 73.95; H, 5.52; N, 9.58. Found: C, 74.21; H, 5.82; N, 9.77.

*4-(Benzo[d]oxazol-2-yl)-1-(p-tolyl)pyrrolidin-2-one* (**14**), pale-yellow solid; yield: 67%; m.p. 164–166 °C; IR: 1411 (C-N), 1562 (C=C), 1598 (C=N), 1697 (C=O), 3077 (Ar C-H); ^1^H-NMR (DMSO-*d*_6_) δ (ppm): 2.28 (s, 3H, CH_3_), 2.94–3.13 (m, 2H, COCH_2_), 4.13–4.35 (m, 3H, NCH_2_ and CH_pyrr_), 7.17–7.20 (d, 2H, H-3_phenyl_ and H-5_phenyl_, *J* = 8.4 Hz), 7.34–7.42 (m, 2H, H-2_phenyl_ and H-6_phenyl_), 7.54–7.57 (d, 2H, H-4_benzoxazole_ and H-7_benzoxazole,_ *J* = 8.4 Hz), 7.70–7.74 (m, 2H, H-5_benzoxazole_ and H-6_benzoxazole_); ESI-MS (*m*/*z*): 292.13 [M]^+^, 293.13 [M + H]^+^; Anal. calcd. for C_18_H_16_N_2_O_2_: C, 73.95; H, 5.52; N, 9.58. Found: C, 74.18; H, 5.78; N, 9.80.

*4-(Benzo[d]oxazol-2-yl)-1-(4-chlorophenyl)pyrrolidin-2-one* (**15**), pale-yellow solid; yield: 70%; m.p. 174–176 °C; IR: 758 (C-Cl), 1396 (C-N), 1568 (C=C), 1612 (C=N), 1689 (C=O), 3086 (Ar C-H); ^1^H-NMR (DMSO-*d*_6_) δ (ppm): 3.01–3.09 (m, 2H, COCH_2_), 4.20–4.33 (m, 3H, NCH_2_ and CH_pyrr_), 7.35–7.43 (m, 4H, Ar-H), 7.68–7.72 (t, 4H, Ar-H, *J* = 6.3 Hz); ESI-MS (*m*/*z*): 312.11 [M]^+^, 314.10 [M + 2]^+^; Anal. calcd. for C_17_H_13_ClN_2_O_2_: C, 65.29; H, 4.19; N, 8.96. Found: C, 65.52; H, 4.35; N, 9.03.

*4-(Benzo[d]oxazol-2-yl)-1-(3-chloro-4-fluorophenyl)pyrrolidin-2-one* (**16**), pale-yellow solid; yield: 68%; m.p. 187–189 °C; IR: 765 (C-Cl), 1388 (C-N), 1545 (C=C), 1613 (C=N), 1690 (C=O), 3098 (Ar C-H); ^1^H-NMR (DMSO-*d*_6_) δ (ppm): 2.92–3.19 (m, 2H, COCH_2_), 4.13–4.21 (m, 1H, CH_pyrr_), 4.25–4.41 (m, 2H, NCH_2_), 7.41–7.60 (m, 5H, Ar-H), 8.05–8.08 (d, 1H, H-7_benzoxazole_, *J* = 8.1 Hz), 8.11–8.14 (d, 1H, H-4_benzoxazole_, *J* = 8.1 Hz); ESI-MS (*m*/*z*): 330.10 [M]^+^, 332.09 [M + 2]^+^; Anal. calcd. for C_17_H_12_ClFN_2_O_2_: C, 61.73; H, 3.66; N, 8.47. Found: C, 62.07; H, 3.85; N, 8.61.

*4-(Benzo[d]oxazol-2-yl)-1-(4-hydroxyphenyl)pyrrolidin-2-one* (**17**), pale-yellow solid; yield: 62%; m.p. 242–245 °C; IR: 1407 (C-N), 1535 (C=C), 1597 (C=N), 1698 (C=O), 3092 (Ar C-H), 3410 (O-H); ^1^H-NMR (DMSO-*d*_6_) δ (ppm): 2.91–3.20 (m, 2H, COCH_2_), 4.12–4.23 (m, 1H, CH_pyrr_), 4.25–4.39 (m, 2H, NCH_2_), 5.64 (bs, 1H, OH, D_2_O exchangeable), 7.41–7.60 (m, 4H, Ar-H_phenyl_), 7.72–7.77 (t, 2H, H-5_benzoxazole_ and H-6_benzoxazole_, *J* = 7.5 Hz), 7.96–7.99 (d, 1H, H-7_benzoxazole_, *J* = 8.4 Hz), 8.08–8.10 (d, 1H, H-4_benzoxazole_, *J* = 8.4 Hz); ESI-MS (*m*/*z*): 294.12 [M]^+^, 295.12 [M + H]^+^; Anal. calcd. for C_17_H_14_N_2_O_3_: C, 69.38; H, 4.79; N, 9.52. Found: C, 69.66; H, 4.97; N, 9.78.

*4-(Benzo[d]oxazol-2-yl)-1-(4-methoxyphenyl)pyrrolidin-2-one* (**18**), pale-yellow solid; yield: 66%; m.p. 177–179 °C; IR: 1412 (C-N), 1546 (C=C), 1600 (C=N), 1690 (C=O), 3081 (Ar C-H); ^1^H-NMR (DMSO-*d*_6_) δ (ppm): 2.88–3.18 (m, 2H, COCH_2_), 3.67 (s, 3H, OCH_3_), 4.08–4.16 (m, 1H, CH_pyrr_), 4.22–4.39 (m, 2H, NCH_2_), 7.16–7.19 (d, 2H, H-2_phenyl_ and H-6_phenyl_, *J* = 8.4 Hz), 7.42–7.58 (m, 4H, H-5_benzoxazole_, H-6_benzoxazole_, H-3_phenyl_ and H-5_phenyl_), 7.95–7.98 (d, 1H, H-7_benzoxazole_, *J* = 8.4 Hz), 8.09–8.12 (d, 1H, H-4_benzoxazole_, *J* = 8.4 Hz); ESI-MS (*m*/*z*): 308.13 [M]^+^, 309.13 [M + H]^+^; Anal. calcd. for C_18_H_16_N_2_O_3_: C, 70.12; H, 5.23; N, 9.09. Found: C, 70.35; H, 5.38; N, 9.18.

*4-(Benzo[d]oxazol-2-yl)-1-(4-nitrophenyl)pyrrolidin-2-one* (**19**), yellow solid; yield: 62%; m.p. 276–278 °C; IR: 1405 (C-N), 1550 (C=C), 1595 (C=N), 1695 (C=O), 3089 (Ar C-H); ^1^H-NMR (DMSO-*d*_6_) δ (ppm): 2.89–3.15 (m, 2H, COCH_2_), 4.11–4.16 (m, 1H, CH_pyrr_), 4.24–4.37 (m, 2H, NCH_2_), 6.79–6.82 (d, 2H, H-2_phenyl_ and H-6_phenyl_, *J* = 9 Hz), 7.43–7.48 (t, 2H, H-5_benzoxazole_, H-6_benzoxazole_, *J* = 7.5 Hz), 8.01–8.18 (m, 4H, H-4_benzoxazole_, H-7_benzoxazole_, H-3_phenyl_ and H-5_phenyl_); ESI-MS (*m*/*z*): 323.11 [M]^+^, 324.11 [M + H]^+^; Anal. calcd. for C_17_H_13_N_3_O_4_: C, 63.16; H, 4.05; N, 13.00. Found: C, 63.35; H, 4.27; N, 13.23.

*4-(4-(Benzo[d]oxazol-2-yl)-2-oxopyrrolidin-1-yl)benzenesulfonamide* (**20**), yellow solid; yield: 60%; m.p. 227–229 °C; IR: 1416 (C-N), 1564 (C=C), 1607 (C=N), 1704 (C=O), 3095 (Ar C-H); ^1^H-NMR (DMSO-*d*_6_) δ (ppm): 2.91–3.19 (m, 2H, COCH_2_), 4.15–4.22 (m, 1H, CH_pyrr_), 4.25–4.38 (m, 2H, NCH_2_), 5.67 (s, 2H, SO_2_NH_2_, D_2_O exchangeable), 7.42–7.59 (m, 4H, Ar-H), 8.09–8.16 (m, 4H, Ar-H); ESI-MS (*m*/*z*): 357.12 [M]^+^, 358.12 [M + H]^+^; Anal. calcd. for C_17_H_15_N_3_O_4_S: C, 57.13; H, 4.23; N, 11.76. Found: C, 57.36; H, 4.36; N, 11.95.

### 4.2. Human MAGL Assay

The screening of the synthesized compounds (**11**–**20**) for their capability to reduce *h*MAGL activity was performed according to the information leaflet provided with Cayman’s assay kit (Cayman Chemical, Michigan, USA) by the reported method [29], as discussed in detail in our previous publication [45]. The results were compared with standard MAGL inhibitors, CAY10499 and JZL184, and are provided in Table 1. 

### 4.3. Human FAAH Assay

The screening of the selected compounds (**15**, **16**, **18**, **19** and **20**) for their potential to inhibit *h*FAAH was performed according to the information leaflet provided with Cayman’s assay kit (Cayman Chemical, Ann Arbor, Michigan, USA) by the reported method [47], as discussed in detail in our previous publication [45]. The results were compared with standard FAAH inhibitor, URB597, and are provided in Table 1. 

### 4.4. Molecular Docking Study 

Glide executed on Maestro 9.4 (Schrödinger Inc., New York, NY, USA) was utilized for Glide XP docking of active compounds (**19** and **20**). The .pdb file of *h*MAGL X-ray crystal structure was downloaded from protein data bank having ID 5ZUN (crystal structure resolution 1.35 Å) for molecular docking study [36]. The protein structure was refined, optimized, and energy-minimized with the help of preparation wizard in Maestro. A docking grid of 20 × 20 × 20 Å, was created around the catalytic site by defining the cocrystallized ligand. Ligand (compounds **19** and **20**) structures were prepared with the help of LigPrep 2.6 with Epik 2.4 at pH 7.0 ± 2.0. The methodology was validated by docking the cocrystallized ligand with Glide XP docking protocol [55].

### 4.5. Physicochemical and Pharmacokinetic Characteristics 

Guidelines, concerning the validation and optimization of orally active CNS compounds, were developed by Ghose et. al. by analyzing 35 characteristic features of orally bioavailable 317 CNS and 626 non-CNS drugs [49]. For computations of these properties of the selected compounds (**19** and **20**), QikProp 3.6 module of Schrodinger was utilized. The generated data was then matched with the qualifying range as per the suggested guideline for good orally bioactive CNS drugs.

### 4.6. In Silico Absorption and Toxicity Profile

The selected compounds (**19** and **20**) were evaluated for their absorption and toxicity profile by a bioinformatics tool admetSAR [50]. Oral bioavailability, intestinal absorption, and BBB penetration properties were calculated. AMES test for mutagenicity, carcinogenicity test, and the calculation of LD50 for both the compounds (**19** and **20**) were also evaluated.

### 4.7. Analgesic Activity

Formalin-induced analgesic test was executed by the procedure described by Coderre and Laughlin [51,52] as discussed in detail in our previous publication [45]. Male Wistar rats (180–200 g) were obtained with the permission of IAEC (proposal number 1048) from Jamia Hamdard, New Delhi, India. The results of test compound **20** and reference drug, Gabapentin (GBP), were statistically compared with the control group.

### 4.8. Anticancer Screening: Sulforhodamine B Assay

Compounds **19** and **20** were supplied to National Cancer Institute (Bethesda, Maryland, USA), for in vitro sulforhodamine B (SRB) assay, anticancer screening on 60 cell lines of cancers of leukemia, melanoma, and tumors of the kidney, brain, breast, lung, colon, ovary, and prostate, as per their standard protocol [53,54]. One dose anticancer results (NCI, USA) of compounds **19** and **20** are provided in the Supplementary Materials.

### 4.9. Statistical Analysis

The statistical study of the data was accomplished by GraphPad Prism (version 8.0.2; GraphPad Software, San Diego, CA, USA). The dose response of the test compounds was compared with that of control, in formalin-induced analgesic test, by analysis of variance (ANOVA) followed by Dunnett’s test. Outcomes are communicated as mean ± SEM.

## 5. Conclusions

Ten benzoxazole clubbed 2-pyrrolidinone derivatives (**11**–**20**) as the inhibitors of MAGL were designed, synthesized, characterized, and assayed against MAGL and FAAH enzymes, in order to find potential small molecule selective MAGL inhibitor. Compounds **19** (4-NO_2_ derivative) and **20** (4-SO_2_NH_2_ derivative) were found most potent and selective, with an IC_50_ of 8.4 and 7.6 nM, respectively. The binding patterns of compounds **19** and **20** in the catalytic site of MAGL were found as expected and similar as those of the reported inhibitors.

The physiochemical and pharmacokinetic properties of compounds **19** and **20** were found to be almost in the qualifying range as per the proposed guideline for good orally bioactive CNS drugs. Compound **20** significantly demonstrated the reduction in pain response, having better potency than the positive control GBP, at the dose of 30 mg/kg. The present work concluded that compound **20** is the potential lead compounds that can be further studied and optimized at points 1 and 4 of the 2-pyrrolidinone moiety for the discovery and development of more selective and potent inhibitors of MAGL for neuropathic pain. 

## Figures and Tables

**Table 1:** In vitro *h*MAGL and *h*FAAH inhibition assay of the synthesized compounds (**11**–**20**).

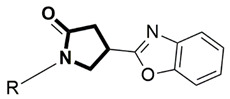			
Compound	R	*h*MAGL; IC_50_	*h*FAAH; IC_50_
**11**	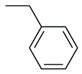	>100 μM	ND
**12**	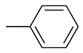	>100 μM	ND
**13**	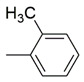	85 ± 1.5 μM	ND
**14**	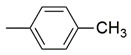	72 ± 2.1 μM	ND
**15**	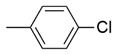	65 ± 3.2 nM	28 ± 1.8 μM
**16**	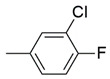	34 ± 1.7 nM	25 ± 2.3 μM
**17**	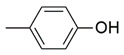	62 ± 2.7 μM	ND
**18**	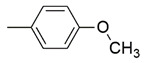	42 ± 1.5 nM	37 ± 2.2 μM
**19**	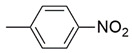	8.4 ± 1.9 nM	55 ± 2.7 μM
**20**	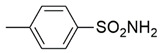	7.6 ± 0.8 nM	68 ± 2.1 μM
**CAY10499**	--	415 ± 3.2 nM	--
**JZL184**	--	10 ± 0.8 nM	--
**URB597**	--	--	5 ± 0.6 nM

ND: Not determined; CAY10499, JZL184 and URB597 (standard control); IC_50_ values were calculated from GraphPad Prism (ver. 8.0.2). Results are expressed as mean ± SEM (*n* = 3).

**Table 2:** Physicochemical and pharmacokinetic properties of compounds **19** and **20**, predicted by QikProp, Schrodinger, for CNS activity.

S. No.	Property	Description	Range of Properties in CNS Drugs				Compound 19	Compound 20
			QL	PL	PU	QU		
1	#stars	drug likeness penalty; the higher the value, the less drug-like the molecule	0	0	0	3	0	0
2	#amine	no. of basic amines	0	1	1	2	0	0
3	#amidine	no. of amidines groups	0	0	0	0	0	0
4	#acid	no. of carboxylic acid groups	0	0	0	0	0	0
5	#amide	no. of amides groups	0	0	0	1	0	0
6	#rotor	no. of rotatable bonds (without CX3, alkene, amide, small ring)	0	3	6	8	1	2
7	CNS	a qualitative CNS activity parameter	−2	0	1	2	−2	−2
8	dipole	computed dipole moment	0.67	1.1	3.9	8.9	9.47	10.22
9	SASA	solvent accessible surface area	348	487	620	798	584.85	617.51
10	FOSA	SASA on saturated carbon and attached hydrogen	16	178	314	464	91.99	91.96
11	FISA	SASA on N, O, and H attached to heteroatoms	0	0	64	176	167.04	210.06
12	PISA	π component of SASA	0	160	292	343	325.81	313.57
13	WPSA	weakly polar component of the SASA (halogens, P, and S)	0	0	0	126	0	1.94
14	volume	solvent accessible volume (Å^3^)	492	830	1104	1388	1002.52	1065.46
15	donorHB	estimated no. of hydrogen bonds that would be donated to the solvent water	0	0	1	3	0	2
16	accptHB	estimated no. of hydrogen bonds that would be accepted from the solvent water	1	2.8	5.2	8.3	6	9.5
17	glob	a globularity descriptor (1 for a sphere)	0.77	0.82	0.88	0.93	0.82	0.81
18	QPpolrz	predicted polarizability (Å^3^)	14	28	38	49	36.43	38.19
19	QPlogPo/w	octanol−water logP	−0.16	2.5	4.7	6.0	2.13	0.90
20	QPlogS	solubility in log(moles/liter)	−6.5	−4.6	−2.5	−0.42	−3.99	−3.99
21	CIQPlogS	log of conformation-independent solubility	−6.3	−4.2	−2.3	0.36	−4.16	−3.77
22	QPPCaco	apparent Caco-2 cell permeability	0	0	810	3269	258.09	100.92
23	QPlogBB	brain/blood partition coefficient	−1.2	−0.06	0.75	1.2	−1.12	−1.65
24	QPPMDCK	predicted apparent MDCK cell permeability (nm/s)	0	0	634	5899	114.43	42.50
25	QPlogKhsa	prediction of binding to human serum albumin	−1	0.04	0.78	1.04	−0.11	−0.34
26	HumanOralAbsorption	Human oral absorption	2	3	3	3	3	3
27	PercentHuman OralAbsorption	Percent of human oral absorption	61	95	100	100	82.63	68.11
28	TPSA	van der Waals surface area of polar nitrogen and oxygen atoms	3.8	12	54	109	98.65	119.08
29	#NandO	no. of N and O atoms	1	2	4	7	7	7
30	RuleOfFive	no. of violations of Lipinski’s rule of five	0	0	0	1	0	0
31	RuleOfThree	no. of violations of Jorgensen’s rule of three	0	0	0	1	0	0
32	#in34	no. of atoms in three- or four-membered rings	0	0	0	0	0	0
33	#in56	no. of atoms in five- or six-membered rings	5	11	17	24	20	20
34	#noncon	no. of atoms not able to form conjugation in nonaromatic rings	0	0	4	10	3	3
35	#nonHatm	no. of non-H atoms	8	19	25	30	24	25

Abbreviations: QL, qualifying lower limit; PL, preferred lower limit; QU, qualifying upper limit; PU, preferred upper limit. # QL, PL, QU and PU values for CNS drug criteria were obtained from reference [49].

**Table 3:** In-silico absorption and toxicity profile of compounds **19** and **20** obtained from admetSAR server [50].

Compound	BBB	HIA	HOB	AMES test	Carcinogenicity	Rat Acute Toxicity (LD50, mol/kg)
19	Yes	Yes	Yes	Mutagenic	Non-carcinogen	2.30
20	Yes	Yes	Yes	Non-Mutagenic	Non-carcinogen	2.21

BBB: blood–brain barrier; HIA: human intestinal permeability; HOB: human oral bioavailability; AMES test is to detect a probable mutagen; carcinogenicity estimates the cancer causing ability of a molecule; LD50: lethal dose which could kill 50% of the population of the organism (rat) on which it is being tested.

**Table 4:** In vitro anticancer screening of compound **19** and **20**, against NCI60 cell lines at 10 μM concentration.

Panel	Cell Line	Compound 19 (NSC: 778839)		Compound 20 (NSC: 778842)	
		% G	% GI	% G	% GI
Leukemia	CCRF-CEM	93.58	6.42	94.48	5.52
	HL-60(TB)	100.09	−0.09	96.15	3.85
	K-562	98.41	1.59	98.83	1.17
	MOLT-4	93.64	6.36	92.47	7.53
	RPMI-8226	101.28	−1.28	103.53	−3.53
	SR	88.92	11.08	93.20	6.80
Non-Small Cell Lung Cancer	A549/ATCC	100.60	−0.60	95.28	4.72
	HOP-62	85.04	14.96	81.97	18.03
	HOP-92	104.72	−4.72	77.78	22.22
	NCI-H226	98.63	1.37	92.75	7.25
	NCI-H23	93.07	6.93	92.19	7.81
	NCI-H322M	94.18	5.82	99.10	0.90
	NCI-H460	102.33	−2.33	104.20	−4.20
Colon Cancer	COLO 205	103.79	−3.79	104.05	−4.05
	HCC-2998	102.05	−2.05	100.24	−0.24
	HCT-116	102.14	−2.14	95.78	−4.22
	HCT-15	98.10	1.9	101.16	−1.16
	HT29	99.25	0.75	103.23	−3.23
	KM12	105.43	−5.43	101.16	−1.16
	SW-620	102.52	−2.52	102.95	−2.95
CNS Cancer	SF-268	91.85	8.15	87.43	12.57
	SF-295	98.21	1.79	93.71	6.29
	SF-539	95.59	4.41	87.58	12.42
	SNB-19	99.29	0.71	97.02	2.98
	SNB-75	64.51	35.49	68.12	31.88
	U251	100.97	−0.97	95.21	4.79
Melanoma	LOX IMVI	89.06	10.94	92.99	7.01
	MALME-3M	88.53	11.47	93.29	6.71
	M14	101.37	−1.37	98.58	1.42
	MDA-MB-435	95.05	4.95	100.55	−0.55
	SK-MEL-2	102.31	−2.31	111.54	−11.54
	SK-MEL-28	111.25	−11.25	101.79	−1.79
	SK-MEL-5	98.72	1.28	98.82	−1.18
	UACC-257	106.92	−6.92	110.78	−10.78
	UACC-62	97.59	2.41	92.78	7.22
Ovarian Cancer	IGROV1	104.14	−4.14	101.63	−1.63
	OVCAR-3	98.56	1.44	98.53	1.47
	OVCAR-4	106.27	−6.27	99.47	0.53
	OVCAR-5	98.30	1.70	92.08	7.92
	OVCAR-8	101.95	−1.95	97.49	2.51
	NCI/ADR-RES	98.37	1.63	101.45	−1.45
	SK-OV-3	88.34	11.66	94.77	5.23
Renal Cancer	786-0	104.06	−4.06	98.99	1.01
	A498	113.46	−13.46	113.94	−13.94
	ACHN	91.70	8.3	89.48	10.52
	CAKI-1	97.19	2.81	92.35	7.65
	SN12C	97.21	2.79	95.50	4.50
	TK-10	110.14	−10.14	114.82	−14.82
	UO-31	78.82	21.18	70.05	29.95
Prostate Cancer	PC-3	91.14	8.86	88.11	11.89
	DU-145	110.09	−10.09	111.62	−11.62
Breast Cancer	MCF7	99.19	0.81	92.32	7.68
	MDA-MB-231/ATCC	88.41	11.59	80.11	19.89
	HS 578T	101.81	−1.81	104.19	−4.19
	T-47D	80.01	19.99	83.24	16.24
	MDA-MB-468	98.03	1.97	100.34	−0.34

## Data Availability

The data presented in this study are available on request from the corresponding authors.

## Supplementary Materials

The following are available online, ^1^H-NMR spectra of compounds **11**–**20** and one dose anticancer results (NCI, USA) of compounds **19** and **20** is provided in the supporting information.

## Sample Availability

The samples of the compounds are available on request from the corresponding author.

## References

[1] Di Marzo V., Bisogno T., De Petrocellis L. (2005). the biosynthesis, fate and pharmacological properties of endocannabinoids. Handb. Exp. Pharmacol..

[2] Sugiura T., Kondo S., Sukagawa A., Nakane S., Shinoda A., Itoh K. (1995). 2- Arachidonoylglycerol: A possible endogenous can-nabinoid receptor ligand in brain. Biochem. Biophys. Res. Commun..

[3] Matias I., Bisogno T., Di Marzo V., Endocannabinoid Research Group (2006). Endogenous cannabinoids in the brain and peripheral tissues: Regulation of their levels and control of food intake. Int. J. Obes..

[4] Blankman J.L., Simon G.M., Cravatt B.F. (2007). A comprehensive profile of brain enzymes that hydrolyze the endocannabinoid 2-arachidonoylglycerol. Chem. Biol..

[5] Savinainen J.R., Saario S.M., Laitinen J.T. (2011). The serine hydrolases MAGL, ABHD6 and ABHD12 as guardians of 2-arachidonoylglycerol signalling through cannabinoid receptors. Acta Physiol..

[6] Kinsey S.G., Long J.Z., O’Neal S.T., Abdullah R.A., Poklis J.L., Boger D., Cravatt B.F., Lichtman A.H. (2009). Blockade of endo-cannabinoid-degrading enzymes attenuates neuropathic pain. J. Pharmacol. Exp. Ther..

[7] Kinsey S.G., Long J.Z., Cravatt B.F., Lichtman A.H. (2010). Fatty acid amide hydrolase and monoacylglycerol lipase inhibitors produce anti-allodynic effects in mice through distinct cannabinoid receptor mechanisms. J. Pain.

[8] Hohmann A.G., Suplita R.L., Bolton N.M., Neely M.H., Fegley D., Mangieri R., Krey J.F., Walker J.M., Holmes P.V., Crystal J.D. (2005). An endocannabinoid mechanism for stress-induced analgesia. Nat. Cell Biol..

[9] Connell K., Bolton N., Olsen D., Piomelli D., Hohmann A.G. (2006). Role of the basolateral nucleus of the amygdala in endocan-nabinoid-mediated stress-induced analgesia. Neurosci. Lett..

[10] Guindon J., Guijarro A., Piomelli D., Hohmann A.G. (2010). Peripheral antinociceptive effects of inhibitors of monoacylglycerol lipase in a rat model of inflammatory pain. Br. J. Pharmacol..

[11] Guindon J., Lai Y., Takacs S.M., Bradshaw H.B., Hohmann A.G. (2013). Alterations in endocannabinoid tone following chemotherapy-induced peripheral neuropathy: Effects of endocannabinoid deactivation inhibitors targeting fatty-acid amide hydrolase and monoacylglycerol lipase in comparison to reference analgesics following cisplatin treatment. Pharmacol. Res..

[12] Aaltonen N., Kedzierska E., Orzelska-Górka J., Lehtonen M., Navia-Paldanius D., Jakupovic H., Savinainen J.R., Neva-lainen T., Laitinen J.T., Parkkari T. (2016). In vivo characterization of the ultrapotent monoacylglycerol lipase inhibitor {4-[bis-(benzo[d][1,3]dioxol-5-yl)methyl]-piperidin-1-yl}(1H-1,2,4-triazol-1-yl)methanone (JJKK-048). J. Pharm. Exp. Ther..

[13] Nomura D.K., Lombardi D.P., Chang J.W., Niessen S., Ward A.M., Long J.Z., Hoover H.H., Cravatt B.F. (2011). Monoacylglycerol lipase exerts dual control over endocannabinoid and fatty acid pathways to support prostate cancer. Chem. Biol..

[14] Chen R., Zhang J., Wu Y., Wang D., Feng G., Tang Y.-P., Teng Z., Chen C. (2012). Monoacylglycerol lipase is a therapeutic target for Alzheimer’s Disease. Cell Rep..

[15] McAllister L.A., Butler C.R., Mente S., O’Neil S.V., Fonseca K.R., Piro J.R., Cianfrogna J.A., Foley T.L., Gilbert A.M., Harris A.R. (2018). Discovery of trifluoromethyl glycol carbamates as potent and selective covalent monoacylglycerol lipase (MAGL) inhibitors for treatment of neuroinflammation. J. Med. Chem..

[16] Cisar J.S., Weber O.D., Clapper J.R., Blankman J.L., Henry C.L., Simon G.M., Alexander J.P., Jones T.K., Ezekowitz R.A.B., O’Neill G.P. (2018). Identification of ABX-1431, a selective inhibitor of monoacylglycerol lipase and clinical candidate for treatment of neurological disorders. J. Med. Chem..

[17] Maione S., Morera E., Marabese I., Ligresti A., Luongo L., Ortar G., DiMarzo V. (2008). Antinociceptive effects of tetrazole inhib-itors of endocannabinoid inactivation: Cannabinoid and noncannabinoid receptor-mediated mechanisms. Br. J. Pharmacol..

[18] Fowler C.J. (2012). Monoacylglycerol lipase—A target for drug development?. Br. J. Pharmacol..

[19] Mulvihill M.M., Nomura D.K. (2013). Therapeutic potential of monoacylglycerol lipase inhibitors. Life Sci..

[20] Alhouayek M., Masquelier J., Muccioli G.G. (2014). Controlling 2-arachidonoylglycerol metabolism as an anti-inflammatory strategy. Drug Discov. Today.

[21] Guzman M. (2010). A new age for MAGL. Chem. Biol..

[22] Long J.Z., Nomura D.K., Cravatt B.F. (2009). Characterization of monoacylglycerol lipase inhibition reveals differences in central and peripheral endocannabinoid metabolism. Chem. Biol..

[23] Lass A., Zimmermann R., Oberer M., Zechner R. (2011). Lipolysis—A highly regulated multi-enzyme complex mediates the catabolism of cellular fat stores. Prog. Lipid Res..

[24] Dorsam R.T., Gutkind J.S. (2007). G-protein-coupled receptors and cancer. Nat. Rev. Cancer.

[25] Nomura D.K., Long J.Z., Niessen S., Hoover H.S., Ng S.-W., Cravatt B.F. (2010). Monoacylglycerol lipase regulates a fatty acid network that promotes cancer pathogenesis. Cell.

[26] Nomura D.K., Morrison B.E., Blankman J.L., Long J.Z., Kinsey S.G., Marcondes M.C.G., Ward A.M., Hahn Y.K., Lichtman A.H., Conti B. (2011). Endocannabinoid hydrolysis generates brain prostaglandins that promote neuroinflammation. Science.

[27] Ye L., Zhang B., Seviour E.G., Tao K.-X., Liu X.-H., Ling Y., Chen J.-Y., Wang G.-B. (2011). Monoacylglycerol lipase (MAGL) knockdown inhibits tumor cells growth in colorectal cancer. Cancer Lett..

[28] King A.R., Duranti A., Tontini A., Rivara S., Rosengarth A., Clapper J.R., Astarita G., Geaga J.A., Luecke H., Mor M. (2007). URB602 inhibits monoacylglycerol lipase and selectively blocks 2-arachidonoylglycerol degradation in intact brain slices. Chem. Biol..

[29] Muccioli G.G., LaBar G., Lambert D.M. (2008). CAY10499, a novel monoglyceride lipase inhibitor evidenced by an expeditious MGL assay. ChemBioChem.

[30] Minkkilä A., Savinainen J.R., Käsnänen H., Xhaard H., Nevalainen T., Laitinen J.T., Poso A., Leppänen J., Saario S.M. (2009). Screening of various hormone-sensitive lipase inhibitors as endocannabinoid-hydrolyzing enzyme inhibitors. ChemMedChem.

[31] Long J.Z., Jin X., Adibekian A., Li W., Cravatt B.F. (2010). Characterization of tunable piperidine and piperazine carbamates as inhibitors of endocannabinoid hydrolases. J. Med. Chem..

[32] Bertrand T., Augé F., Houtmann J., Rak A., Vallée F., Mikol V., Berne P., Michot N., Cheuret D., Hoornaert C. (2010). Structural basis for human monoglyceride lipase inhibition. J. Mol. Biol..

[33] Chang J.W., Niphakis M.J., Lum K.M., Cognetta A.B., Wang C., Matthews M.L., Niessen S., Buczynski M.W., Parsons L.H., Cravatt B.F. (2012). Remarkably selective inhibitors of monoacylglycerol lipase bearing a reactive group that is bioisosteric with endocannabinoid substrates. Chem. Biol..

[34] Morera L., LaBar G., Ortar G., Lambert D.M. (2012). Development and characterization of endocannabinoid hydrolases FAAH and MAGL inhibitors bearing a benzotriazol-1-yl carboxamide scaffold. Bioorg. Med. Chem..

[35] Aaltonen N., Savinainen J.R., Ribas C.R., Rönkkö J., Kuusisto A., Korhonen J., Navia-Paldanius D., Häyrinen J., Takabe P., Käsnänen H. (2013). Piperazine and piperidine triazole ureas as ultrapotent and highly selective inhibitors of monoacylglycerol lipase. Chem. Biol..

[36] Aida J., Fushimi M., Kusumoto T., Sugiyama H., Arimura N., Ikeda S., Sasaki M., Sogabe S., Aoyama K., Koike T. (2018). Design, synthesis, and evaluation of piperazinyl pyrrolidin-2-ones as a novel series of reversible monoacylglycerol lipase inhibitors. J. Med. Chem..

[37] Granchi C., Lapillo M., Glasmacher S., Bononi G., Licari C., Poli G., El Boustani M., Caligiuri I., Rizzolio F., Gertsch J. (2019). Optimization of a benzoylpiperidine class identifies a highly potent and selective reversible monoacylglycerol lipase (MAGL) inhibitor. J. Med. Chem..

[38] Castelli R., Scalvini L., Vacondio F., Lodola A., Anselmi M., Vezzosi S., Carmi C., Bassi M., Ferlenghi F., Rivara S. (2020). Benzisothiazolinone derivatives as potent allosteric monoacylglycerol lipase inhibitors that functionally mimic sulfenylation of regulatory cysteines. J. Med. Chem..

[39] LaBar G., Bauvois C., Borel F., Ferrer J.-L., Wouters J., Lambert D.M. (2009). Crystal structure of the human monoacylglycerol lipase, a key actor in endocannabinoid signaling. ChemBioChem.

[40] Schalk-Hihi C., Schubert C., Alexander R., Bayoumy S., Clemente J.C., Deckman I., DesJarlais R.L., Dzordzorme K.C., Flores C.M., Grasberger B. (2011). Crystal structure of a soluble form of human monoglyceride lipase in complex with an inhibitor at 1.35 Å resolution. Protein Sci..

[41] Scalvini L., Piomelli D., Mor M. (2016). Monoglyceride lipase: Structure and inhibitors. Chem. Phys. Lipids.

[42] Afzal O., Kumar S., Kumar R., Firoz A., Jaggi M., Bawa S. (2014). Docking based virtual screening and molecular dynamics study to identify potential monoacylglycerol lipase inhibitors. Bioorg. Med. Chem. Lett..

[43] Afzal O., Akhtar S., Kumar S., Ali R., Jaggi M., Bawa S. (2016). Hit to lead optimization of a series of N-[4-(1,3-benzothiazol-2-yl)phenyl]acetamides as monoacylglycerol lipase inhibitors with potential anticancer activity. Eur. J. Med. Chem..

[44] Ali M.R., Kumar S., Shalmali N., Afzal O., Azim S., Chanana D., Alam O., Paudel Y.N., Sharma M., Bawa S. (2019). Development of thiazole-5-carboxylate derivatives as selective inhibitors of monoacylglycerol lipase as target in cancer. Mini Rev. Med. Chem..

[45] Altamimi A.S.A., Bawa S., Athar F., Hassan Q., Riadi Y., Afzal O. (2020). Pyrrolidin-2-one linked benzofused heterocycles as novel small molecule monoacylglycerol lipase inhibitors and antinociceptive agents. Chem. Biol. Drug Des..

[46] Mickevicius M., Beresnevicius Z.J., Mickevicius V., Mikulskiene G. (2006). Condensation products of 1-aryl-4-carboxy-2-pyrroli-dinones with o-diaminoarenes, o-aminophenol, and their structural studies. Heteroat. Chem..

[47] Granchi C., Rizzolio F., Palazzolo S., Carmignani S., Macchia M., Saccomanni G., Manera C., Martinelli A., Minutolo F., Tuccinardi T. (2016). Structural optimization of 4-chlorobenzoylpiperidine derivatives for the development of potent, reversible, and selective monoacylglycerol lipase (MAGL) inhibitors. J. Med. Chem..

[48] Mor M., Rivara S., Lodola A., Plazzi P.V., Tarzia G., Duranti A., Tontini A., Piersanti G., Kathuria S., Piomelli D. (2004). Cy-clohexylcarbamic acid 3’- or 4’-substituted biphenyl-3-yl esters as fatty acid amide hydrolase inhibitors: Synthesis, quantitative structure-activity relationships, and molecular modeling studies. J. Med. Chem..

[49] Ghose A.K., Herbertz T., Hudkins R.L., Dorsey B.D., Mallamo J.P. (2012). Knowledge-based, central nervous system (CNS) lead selection and lead optimization for CNS drug discovery. ACS Chem. Neurosci..

[50] Yang H., Lou C., Sun L., Li J., Cai Y., Wang Z., Li W., Liu G., Tang Y. (2018). admetSAR 2.0: Web-service for prediction and optimization of chemical ADMET properties. Bioinformatics.

[51] Coderre T.J., Vaccarino A.L., Melzack R. (1990). Central nervous system plasticity in the tonic pain response to subcutaneous for-malin injection. Brain Res..

[52] Laughlin T.M., Tram K.V., Wilcox G.L., Birnbaum A.K. (2002). Comparison of antiepileptic drugs tiagabine, lamotrigine, and gabapentin in mouse models of acute, prolonged, and chronic nociception. J. Pharmacol. Exp. Ther..

[53] Grever M.R., A Schepartz S., A Chabner B. (1992). The National Cancer Institute: Cancer drug discovery and development program. Semin. Oncol..

[54] Shoemaker R.H. (2006). The NCI60 human tumour cell line anticancer drug screen. Nat. Rev. Cancer.

[55] Friesner R.A., Murphy R.B., Repasky M.P., Frye L.L., Greenwood J.R., Halgren T.A., Sanschagrin P.C., Mainz D.T. (2006). Extra precision glide: Docking and scoring incorporating a model of hydrophobic enclosure for protein-ligand complexes. J. Med. Chem..

