# Supplementary material for: Analgesic and Anticancer Activity of Benzoxazole Clubbed 2-Pyrrolidinones as Novel Inhibitors of Monoacylglycerol Lipase
Source: Molecules. 2021 Apr 20;26(8):2389. doi: 10.3390/molecules26082389 (PMC8074287; doi:10.3390/molecules26082389)
Supplement: Supplementary file 1 [file molecules-26-02389-s001.zip › molecules-1171671-SI.pdf]

Supporting Information

Analgesic and Anticancer Activity of Benzoxazole Clubbed 2-Pyrrolidinones as Novel Inhibitors of Monoacylglycerol Lipase

Obaid Afzal [1,*], Abdulmalik Saleh Alfawaz Altamimi [1,*], Mir Mohammad Shahroz [2], Hemant Kumar Sharma [2], Yassine Riadi [1], and Md Quamrul Hassan [3]

[1] Department of Pharmaceutical Chemistry, College of Pharmacy, Prince Sattam Bin Abdulaziz University, Al Kharj 11942, Saudi Arabia; y.riadi@psau.edu.sa
[2] Department of Pharmaceutical Chemistry, College of Pharmacy, Sri Satya Sai University of Technology and Medical Sciences, Sehore 466001, Madhya Pradesh, India; mirshahroz@gmail.com (M.M.S.); hkspharma@rediffmail.com (H.K.S.)
[3] Department of Pharmacology, School of Pharmaceutical Education and Research, Jamia Hamdard, New Delhi 110062, India; quamrulhassan309@gmail.com
* Correspondence: o.akram@psau.edu.sa (O.A.); as.altamimi@psau.edu.sa (A.S.A.A.); Tel.: +96-611-588-6094 (O.A.); +96-611-588-6072 (A.S.A.A.)

<u>**Table of contents**</u>

S. No.	Content	Page No.
1.	^{1}H NMR spectrum of 4-(benzo[*d*]oxazol-2-yl)-1-benzylpyrrolidin-2-one (**11**)	S3
2.	^{1}H NMR spectrum of 4-(benzo[*d*]oxazol-2-yl)-1-phenylpyrrolidin-2-one (**12**)	S4
3.	^{1}H NMR spectrum of 4-(benzo[*d*]oxazol-2-yl)-1-(o-tolyl)pyrrolidin-2-one (**13**)	S5
4.	^{1}H NMR spectrum of 4-(benzo[*d*]oxazol-2-yl)-1-(p-tolyl)pyrrolidin-2-one (**14**)	S6
5.	^{1}H NMR spectrum of 4-(benzo[*d*]oxazol-2-yl)-1-(4-chlorophenyl)pyrrolidin-2-one (**15**)	S7
6.	^{1}H NMR spectrum of 4-(benzo[*d*]oxazol-2-yl)-1-(3-chloro-4-fluorophenyl)pyrrolidin-2-one (**16**)	S8
7.	^{1}H NMR spectrum of 4-(benzo[*d*]oxazol-2-yl)-1-(4-hydroxyphenyl)pyrrolidin-2-one (**17**)	S9
8.	^{1}H NMR spectrum of 4-(benzo[*d*]oxazol-2-yl)-1-(4-methoxyphenyl)pyrrolidin-2-one (**18**)	S10
9.	^{1}H NMR spectrum of 4-(benzo[*d*]oxazol-2-yl)-1-(4-nitrophenyl)pyrrolidin-2-one (**19**)	S11
10.	^{1}H NMR spectrum of 4-(4-(benzo[*d*]oxazol-2-yl)-2-oxopyrrolidin-1-yl)benzenesulfonamide (**20**)	S12
11.	One dose anticancer results (NCI, USA) of compound **19**	S13
12.	One dose anticancer results (NCI, USA) of compound **20**	S14

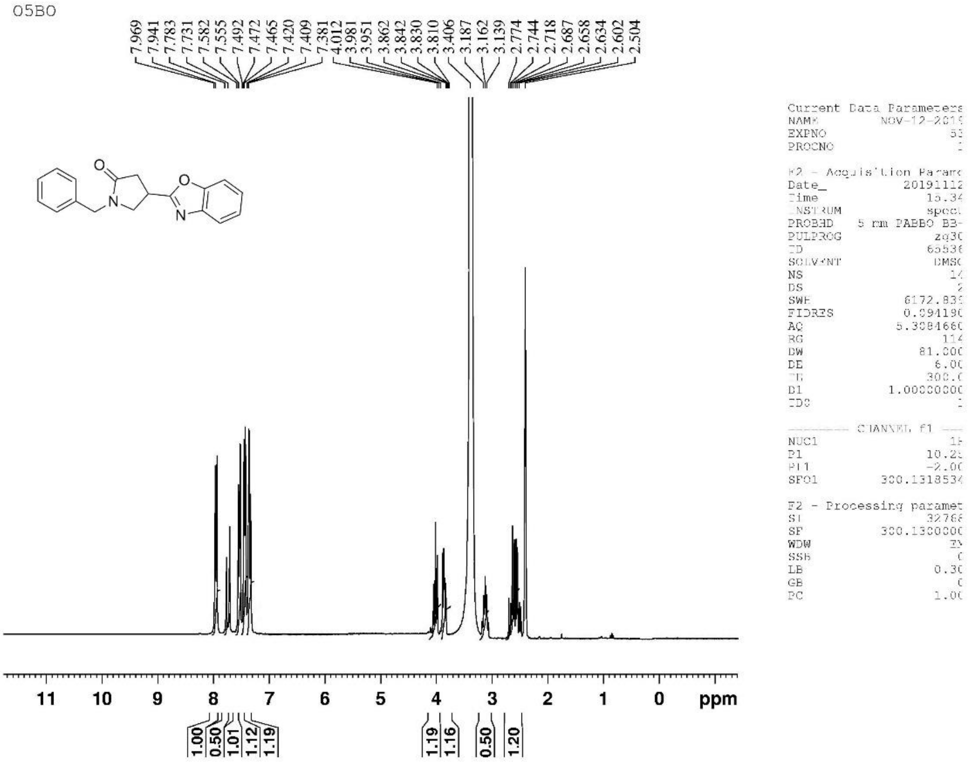

Figure S1: ^{1}H NMR spectrum of 4-(benzo[*d*]oxazol-2-yl)-1-benzylpyrrolidin-2-one (**11**)

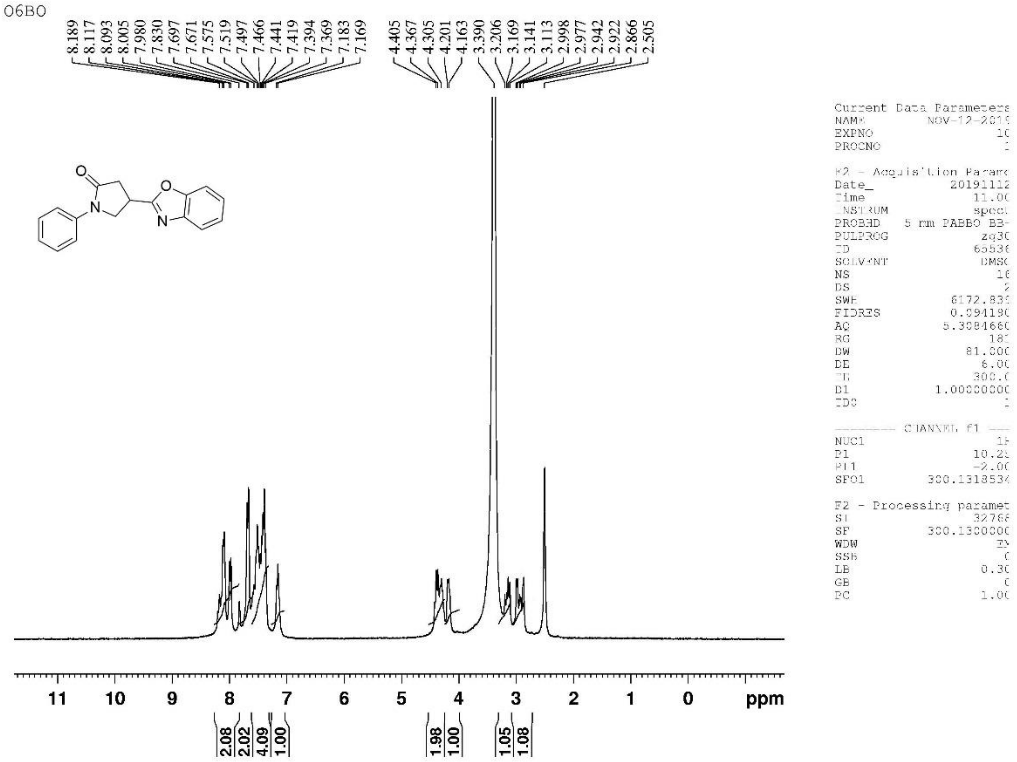

Figure S2: ^{1}H NMR spectrum of 4-(benzo[*d*]oxazol-2-yl)-1-phenylpyrrolidin-2-one (**12**)

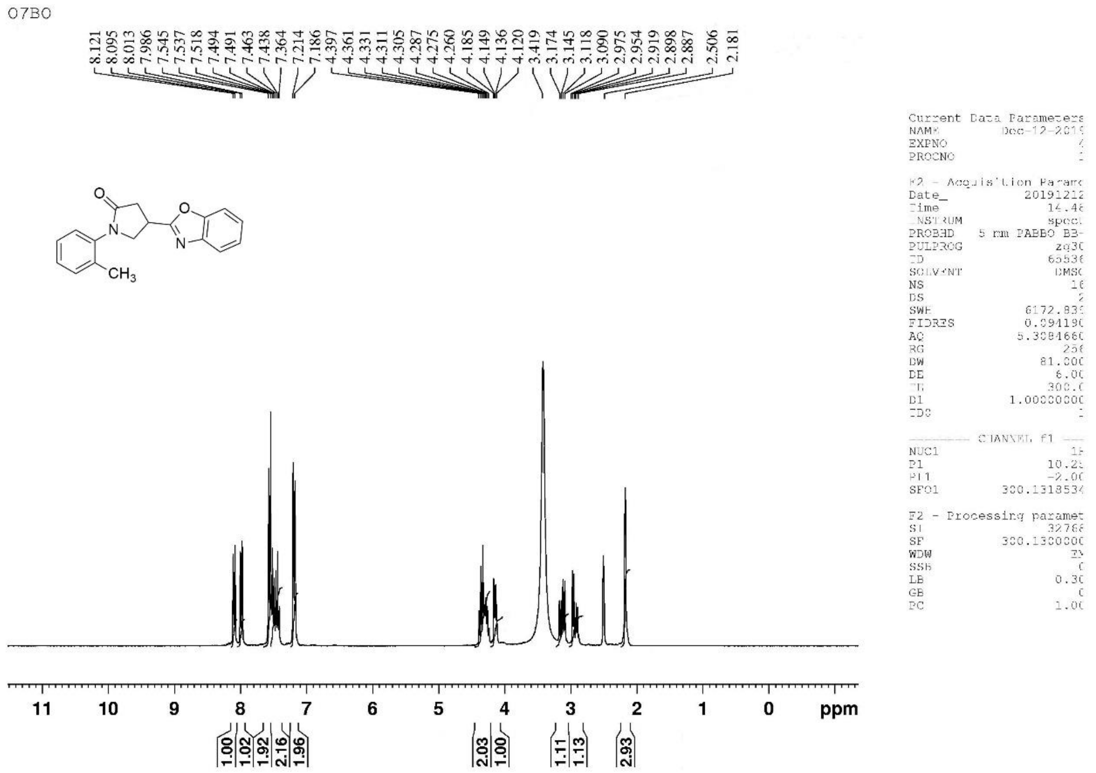

Figure S3: ^{1}H NMR spectrum of 4-(benzo[*d*]oxazol-2-yl)-1-(*o*-tolyl)pyrrolidin-2-one (**13**)

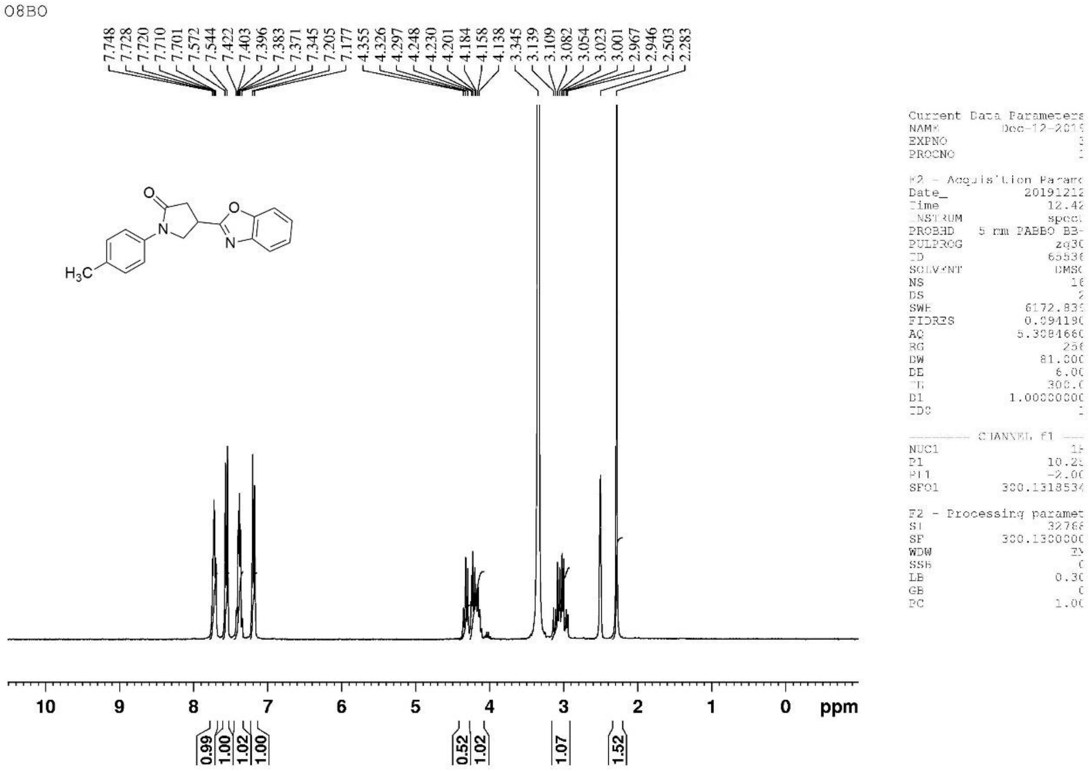

Figure S4: ^{1}H NMR spectrum of 4-(benzo[*d*]oxazol-2-yl)-1-(*p*-tolyl)pyrrolidin-2-one (**14**)

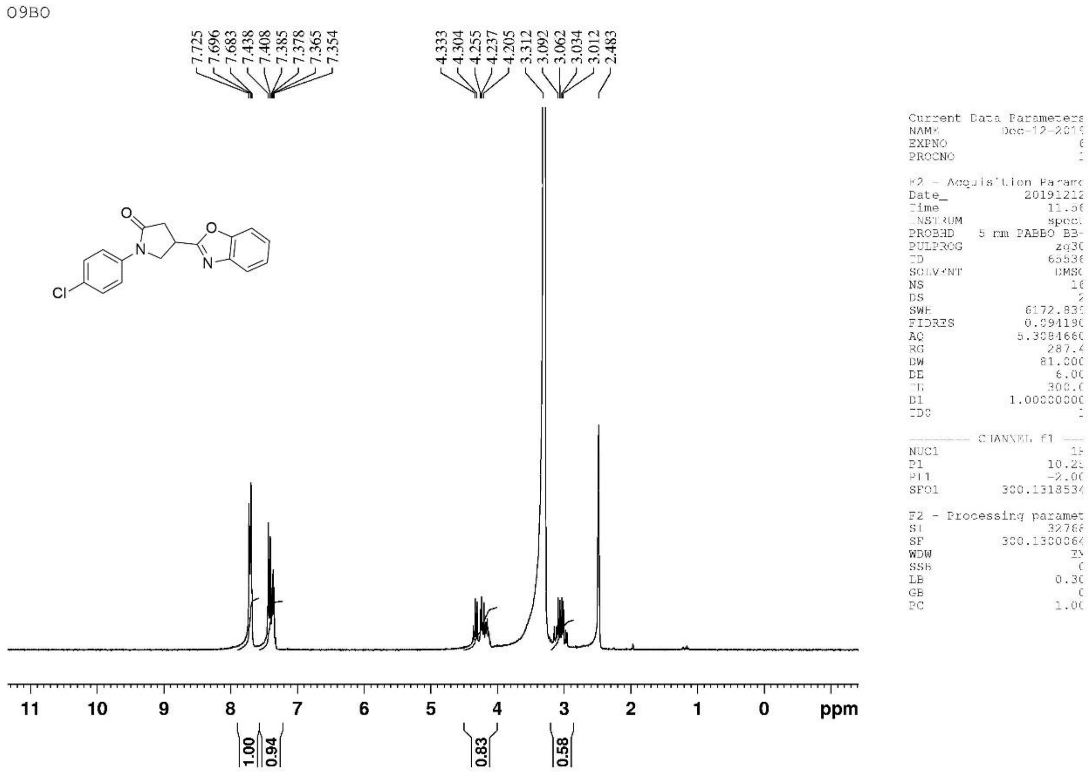

Figure S5: ^{1}H NMR spectrum of 4-(benzo[*d*]oxazol-2-yl)-1-(4-chlorophenyl)pyrrolidin-2-one (**15**)

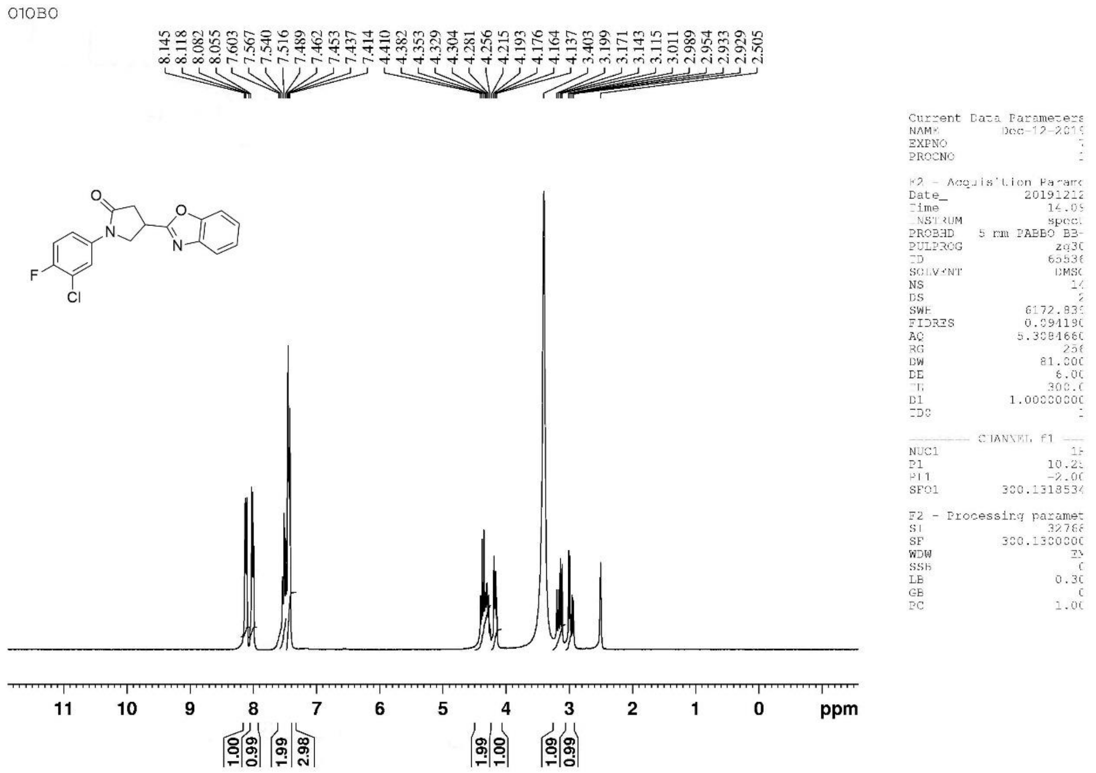

Figure S6: ^{1}H NMR spectrum of 4-(benzo[*d*]oxazol-2-yl)-1-(3-chloro-4-fluorophenyl)pyrrolidin-2-one (**16**)

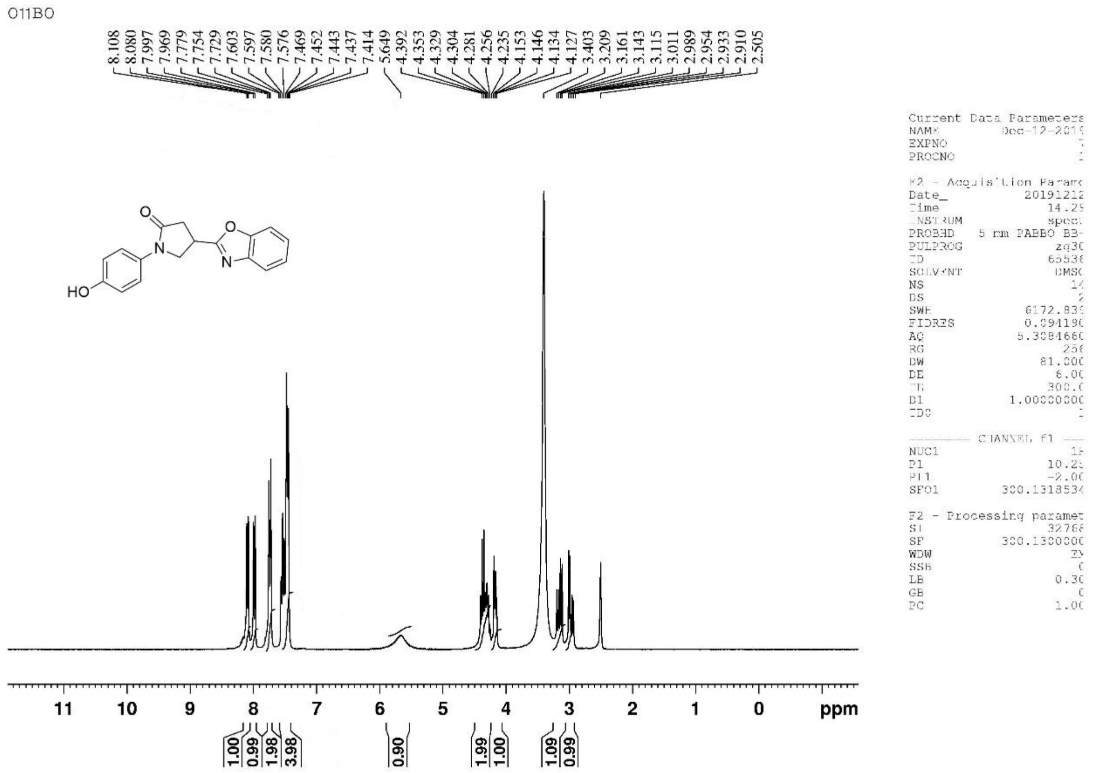

Figure S7: ^{1}H NMR spectrum of 4-(benzo[*d*]oxazol-2-yl)-1-(4-hydroxyphenyl)pyrrolidin-2-one (**17**)

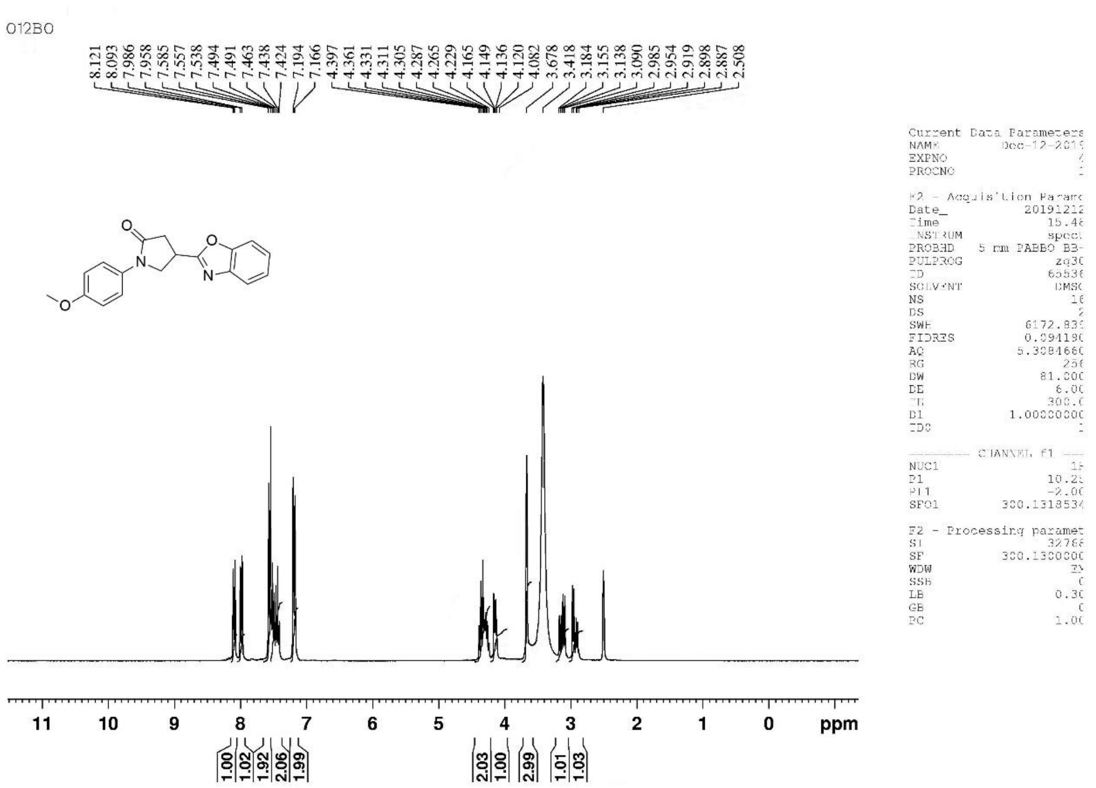

Figure S8: ^{1}H NMR spectrum of 4-(benzo[*d*]oxazol-2-yl)-1-(4-methoxyphenyl)pyrrolidin-2-one (**18**)

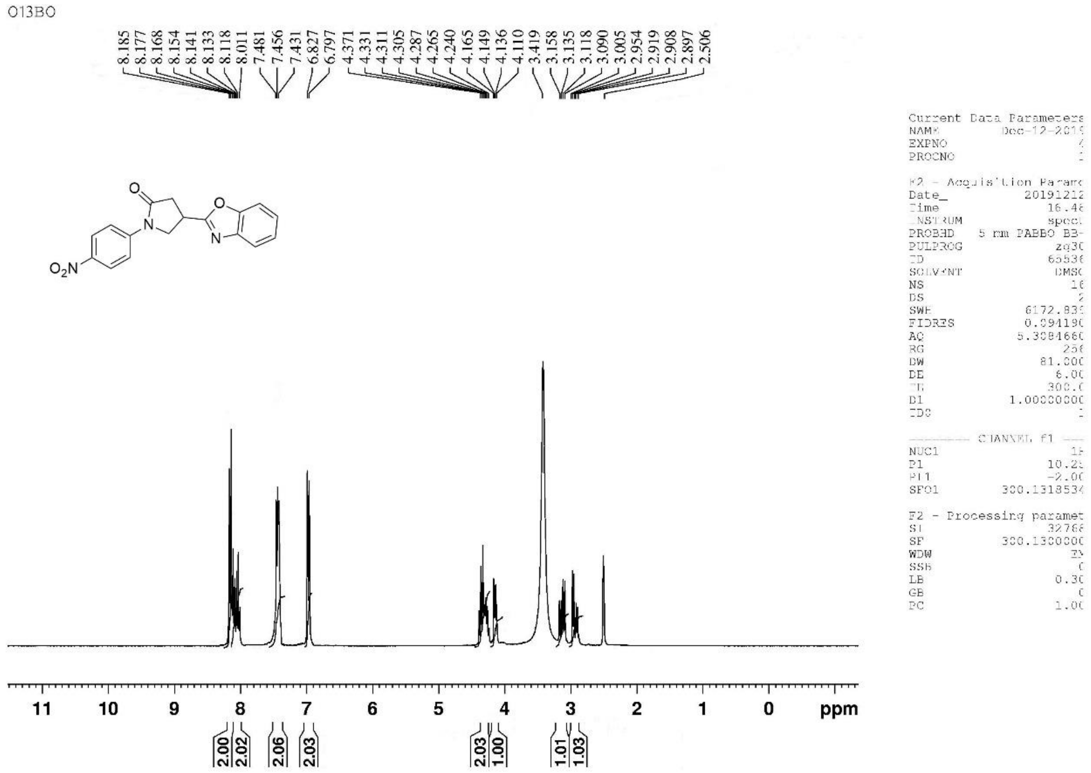

Figure S9: ^{1}H NMR spectrum of 4-(benzo[*d*]oxazol-2-yl)-1-(4-nitrophenyl)pyrrolidin-2-one (**19**)

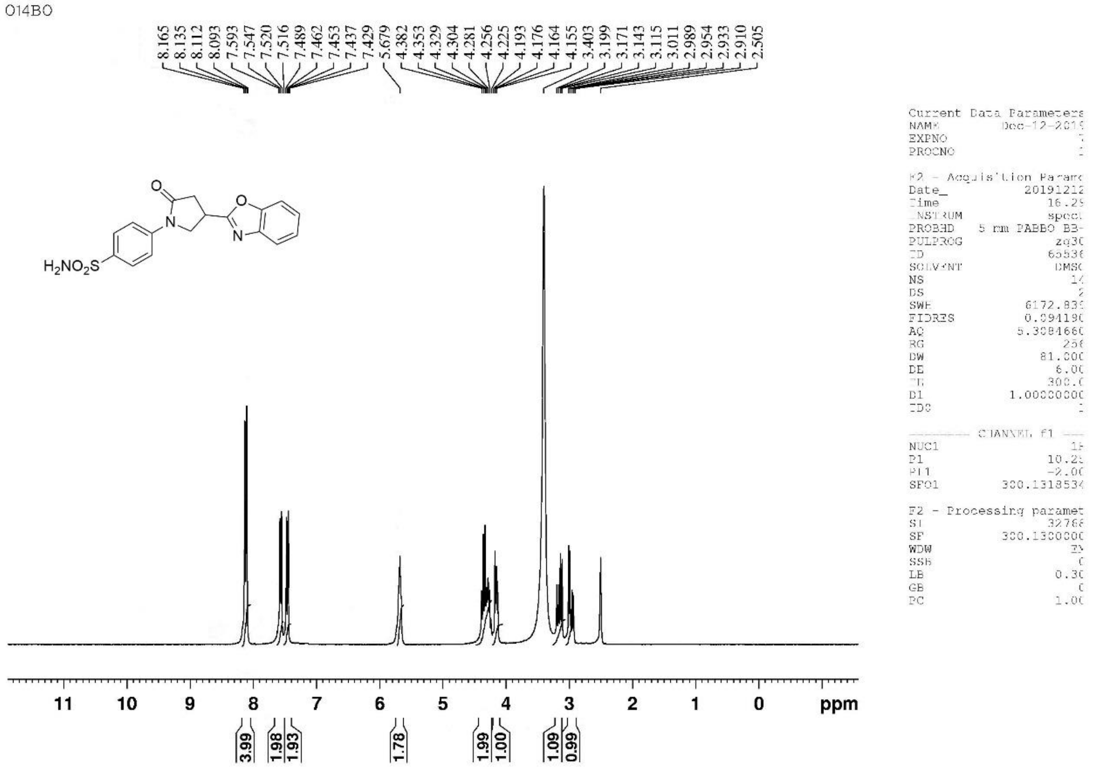

Figure S10: ^{1}H NMR spectrum of 4-(4-(benzo[*d*]oxazol-2-yl)-2-oxopyrrolidin-1-yl)benzenesulfonamide (**20**)

Developmental Therapeutics Program
One Dose Mean Graph

NSC: 778839 / 1 | Conc: 1.00E-5 Molar | Experiment ID: 1401OS23

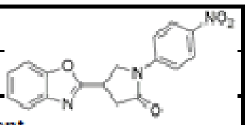

Panel/Cell Line	Growth Percent
**Leukemia**	
CCRF-CEM	93.58
HL-60(TB)	100.09
K-562	98.41
MOLT-4	93.64
RPMI-8226	101.28
SR	88.92
**Non-Small Cell Lung Cancer**	
A549/ATCC	100.60
HOP-62	85.04
HOP-92	104.72
NCI-H226	98.63
NCI-H23	93.07
NCI-H322M	94.18
NCI-H460	102.33
**Colon Cancer**	
COLO 205	103.79
HCC-2998	102.05
HCT-116	102.14
HCT-15	98.10
HT29	99.25
KM12	105.43
SW-620	102.52
**CNS Cancer**	
SF-268	91.85
SF-295	98.21
SF-539	95.59
SNB-19	99.29
SNB-75	64.51
U251	100.97
**Melanoma**	
LOX IMVI	89.06
MALME-3M	88.53
M14	101.37
MDA-MB-435	95.05
SK-MEL-2	102.31
SK-MEL-28	111.25
SK-MEL-5	98.72
UACC-257	106.92
UACC-62	97.59
**Ovarian Cancer**	
IGROV1	104.14
OVCAR-3	98.56
OVCAR-4	106.27
OVCAR-5	98.30
OVCAR-8	101.95
NCI/ADR-RES	98.37
SK-OV-3	88.34
**Renal Cancer**	
786-0	104.06
A498	113.46
ACHN	91.70
CAKI-1	97.19
SN12C	97.21
TK-10	110.14
UO-31	78.82
**Prostate Cancer**	
PC-3	91.14
DU-145	110.09
**Breast Cancer**	
MCF7	99.19
MDA-MB-231/ATCC	88.41
HS 578T	101.81
T-47D	80.01
MDA-MB-468	98.03
Mean	97.61
Delta	33.10
Range	48.95

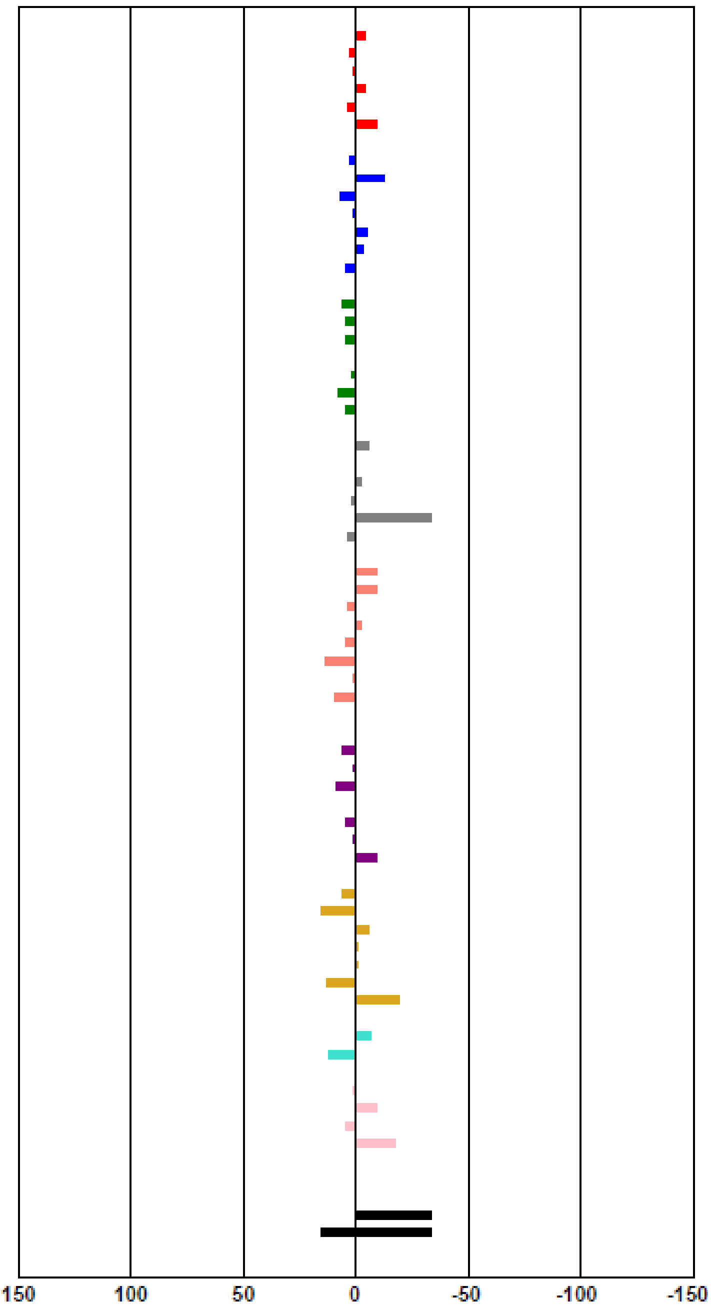

Figure S11: One dose anticancer results (NCI, USA) of compound **19.**

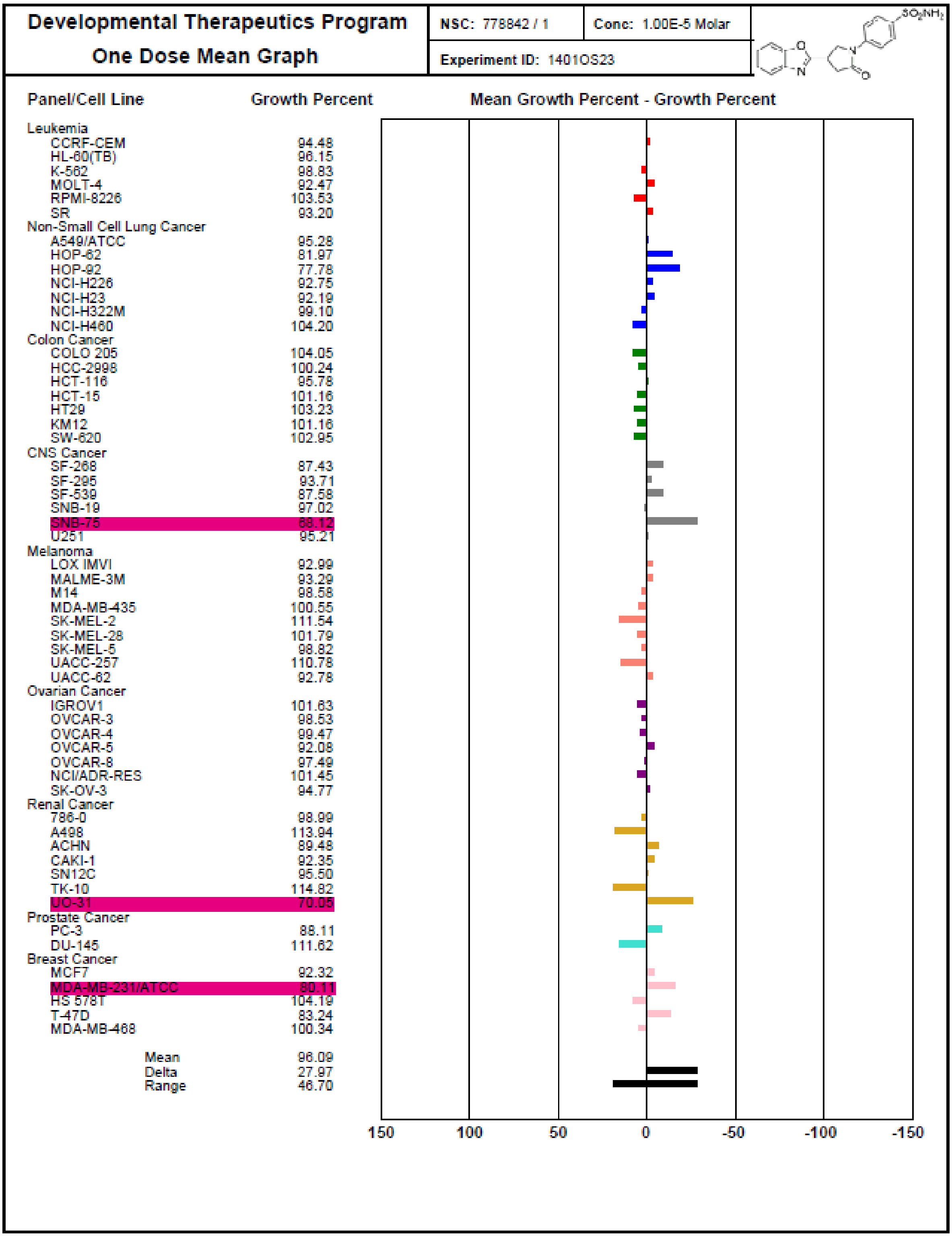

Figure S12: One dose anticancer results (NCI, USA) of compound **20.**
